# Effects of boat traffic and mooring infrastructure on aquatic vegetation: A systematic review and meta-analysis

**DOI:** 10.1007/s13280-019-01215-9

**Published:** 2019-07-11

**Authors:** Josefin Sagerman, Joakim P. Hansen, Sofia A. Wikström

**Affiliations:** 1 The Swedish Species Information Centre (ArtDatabanken), Swedish University of Agricultural Sciences, Box 7007, 750 07 Uppsala, Sweden; 2 Baltic Sea Centre, Stockholm University, 106 91, Stockholm, Sweden

**Keywords:** Boat traffic, Docks, Macrophytes, Marinas, Mooring buoys, Seagrass

## Abstract

Sustainable management of coastal and inland water areas requires knowledge of how tourism and recreation affects the ecosystems. Here, we present the first systematic review and meta-analysis to quantify to what extent recreational boat traffic and infrastructure for mooring affect the abundance of submerged vegetation on soft bottoms. Our systematic search yielded 25 studies containing data on effects of boat traffic, docks and mooring buoys on vegetation abundance. The abundance below docks was on average 18% of that in controls, and areas with boat traffic had on average 42% of the abundance in control areas. Mooring buoys often created scour areas without vegetation. However, the effects were variable and there were too few studies to test the reasons for this variability. We conclude that boating can cause significant declines in submerged vegetation but that informed management of boat traffic and improved design of docks and buoys can reduce negative impacts.

**Electronic supplementary material:**

The online version of this article (10.1007/s13280-019-01215-9) contains supplementary material, which is available to authorized users.

## Introduction

Recreational boating is a popular leisure activity that has increased significantly with economic growth, especially since the mid twentieth century (Hall 2001; Davenport and Davenport 2006; Aall et al. 2011; Burgin and Hardiman 2011). Not only does it offer an opportunity for many people to experience and connect with nature, but it is also important for several local and regional economies (Hassan et al. 2005; Ghermandi and Nunes 2013). However, it may come at a cost to the environment. Recreational boats are often small enough to enter shallow waters, where the effect of boating disturbance can be pronounced, especially in areas with fine sediment bottoms (Klein 1997). Boating is also an important driver for small-scale shoreline exploitation that may have extensive effects on shallow aquatic habitats as a result of cumulative impacts in space and time (Jordan et al. 2009; Sundblad and Bergström 2014; Eriander et al. 2017). Thus, managers of aquatic ecosystems face a potential conflict between promoting recreational use and protecting the ecosystem from adverse effects of recreation. This conflict can for instance arise in the management of protected areas. On the one hand, recreation is regarded as one important benefit from nature conservation and recreational use may increase where protected areas are established (Rees et al. 2015; Gonson et al. 2016). On the other hand, boating can disturb habitats that needs protection (e.g. Liddle and Scorgie 1980; Burgin and Hardiman 2011).

Submerged aquatic vegetation forms ecologically important habitats on soft sediment bottoms in shallow freshwater and coastal ecosystems, providing multiple benefits for biodiversity and human welfare. Meadows of aquatic plants store nutrients and carbon (Wang et al. 2016), acting as a natural filter for nutrients from land (McGlathery et al. 2007) and a significant carbon sink (Fourqurean et al. 2003; Serrano et al. 2016; Wang et al. 2016). The vegetation stabilizes the sediment and decreases sediment resuspension, which results in clear water (Madsen et al. 2001; Scheffer 2004; Austin et al. 2017). The vegetation also provides food and habitat for a large variety of species, from invertebrates to fish, birds and mammals (e.g. Hemminga and Duarte 2000; Scheffer 2004). Aquatic vegetation is threatened by a number of direct and indirect interacting human pressures, such as nutrient and sediment loadings, fishing, shoreline development and physical disturbances—which can result in extensive vegetation declines (Lotze et al. 2006; Orth et al. 2006; Eriksson et al. 2011). Loss of aquatic vegetation can be difficult to reverse due to feed-back mechanisms that either reinforce vegetation dominance or inhibit vegetation recovery, as described for temperate lakes (Scheffer 2004) and coastal seagrass areas (Maxwell et al. 2016; Moksnes et al. 2018). Conservation of aquatic vegetation is therefore a priority for management of freshwater and coastal areas.

**Fig. 1:**
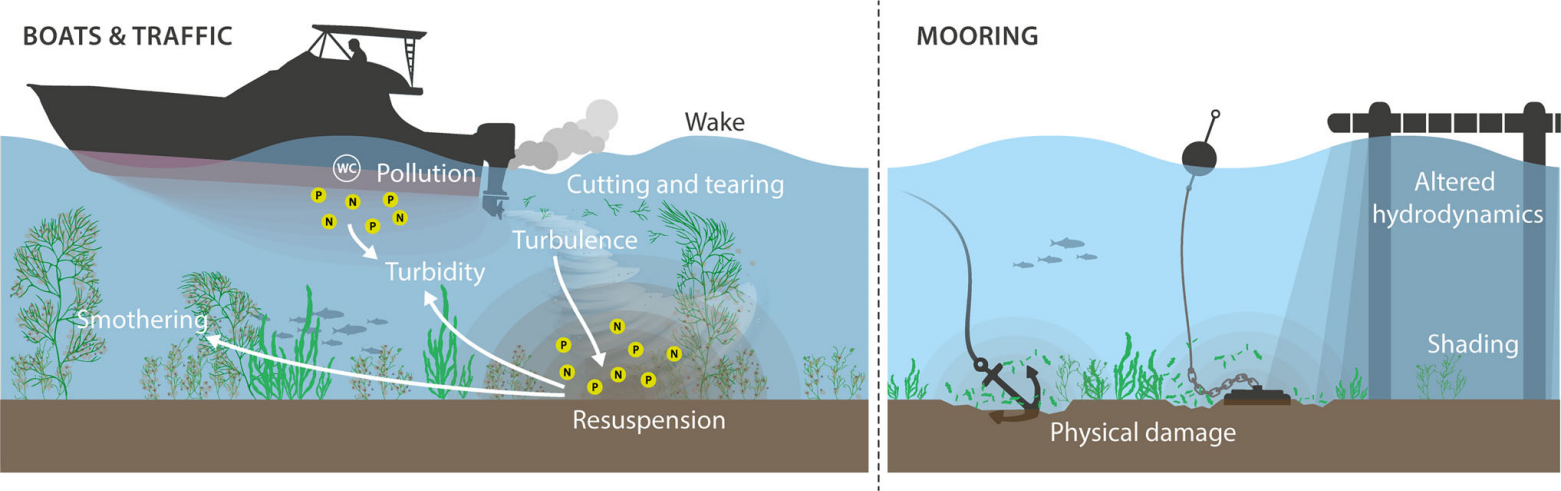
Illustration of mechanisms by which recreational boating activities affect submerged aquatic vegetation, separated into mechanisms generated by boats (left hand side) and mooring facilities (right hand side). Graphics: J. Lokrantz/Azote

The current knowledge on effects of recreational boat traffic on the aquatic environment has been summarized in a number of reviews (e.g. Liddle and Scorgie 1980; Mosisch and Arthington 1998; Burgin and Hardiman 2011) and government reports (Klein 1997; Asplund 2000), showing that boating can have a number of different, potentially interacting, effects on submerged aquatic vegetation (Fig. 1). The propellers of motorboats can directly cut or uproot the vegetation, and sensitive species can be damaged by wake and turbulence generated by propellers and boat movement. Water turbulence and wake also stir up sediment, resulting in shading of benthic vegetation due to increased water turbidity and in smothering when sediment settle on the shoots. The resuspension of sediments can release sediment nutrients, stimulating phytoplankton growth that also results in shading of benthic vegetation. In addition to the physical effects of propellers and increased water movement, recreational boats can contribute to chemical pollution by fuel and lubricants from combustion engines and biocides from anti-fouling paint (e.g. Eklund et al. 2010; Egardt et al. 2018), and to eutrophication due to inadequate wastewater treatment. Beside the effects of boat traffic, anchors, buoys and docks used for mooring of recreational boats can create additional disturbance to aquatic vegetation. Anchoring and mooring buoys create mechanical damage to the vegetation and stir up sediment (e.g. Hastings et al. 1995; Ostendorp et al. 2009; Unsworth et al. 2017). Dock constructions result in shading of the bottom under the dock (Campbell and Baird 2009; Eriander et al. 2017) and can change hydrodynamic conditions, resulting in erosion and translocation of sediment (Dugan et al. 2011).

The emerging conclusion from previous studies and reviews of boating effects is that recreational boating can be a driver of vegetation decline, but the magnitude of the problem is still discussed (e.g. Mosisch and Arthington 1998; Asplund 2000). Individual studies differ in reported impacts, which means that it is difficult to predict the effect in unstudied sites, in particular since the sensitivity to boating disturbance is likely to differ between species and habitats (e.g. Willby et al. 2001; Eriksson et al. 2004; Hansen and Snickars 2014). There has been no systematic review of the existing evidence, attempting to estimate the magnitude of effect across independent studies from different regions or habitats, or testing if the effect differ predictably between for instance biogeographic regions, habitat types or vegetation communities. Such knowledge is crucial for managers that need to balance access for recreation against protection of sensitive species and habitats. Systematic reviews are transparent, repeatable, objective and less biased than traditional reviews and are increasingly used to support policy making in conservation and environmental management (e.g. Haddaway and Pullin 2014).

The aim of this paper is to quantify to what extent recreational boat traffic and mooring facilities impact on the abundance of submerged aquatic vegetation on soft substrate. We perform a systematic review to locate, select and critically appraise relevant data and summarize the data using meta-analysis to get an unbiased quantification of the effect. Initially, we also aimed to test if the effect of recreational boating differ in magnitude depending on boating intensity, depth, biogeographic region, type of environment or vegetation type, but this turned out to be difficult due to limited data. Instead, we identify needs for further research to help establish the factors behind the observed variation in boating impact. Thus, we anticipate that our results will provide an evidence base to aid decision-making, but also help identify and prioritize further research needs.

## Materials and methods

The methods used to locate, select and critically appraise relevant data followed our a priori established review protocol (Appendix S1).

### Search for literature

We searched for relevant studies in the online data bases “ISI Web of Science” and “ASFA: Aquatic Science and Fishery Abstracts” the 30th of November 2015 using the topic or abstract field, with no restriction on publication year or language. In addition, we did a complementary search on 20th of March 2019 using exactly the same method, in order to capture studies published up to this date. We used the following search string: (macrophyte* OR seagrass OR SAV OR Charophyt* OR *Chara* OR Nitell* OR *Thalassia* OR *Posidonia* OR *Halophila* OR *Zostera* OR *Potamogeton* OR *Myriophyllum* OR *Ruppia* OR *Ranunculus* OR *Elodea* OR *Ceratophyllum* OR *Alisma* OR *Hydrilla* OR *Bryophyta* OR *Utricularia* OR Nympha* OR *Nasturtium* OR *Vallisneria* OR *Cymodocea* OR ((aquatic OR benthic OR submerged OR underwater) AND (vegetat* OR plant* OR *flora* OR weed))) AND (*boat* OR *ferry* OR ship* OR watercraft* OR berth* OR mooring* OR anchor* OR wake* OR propeller OR pier OR jetty OR (wave NEAR/1 action)). In order to retrieve studies including boating marinas without getting all publications including the species *Zostera marina*, we performed an additional search using the search string as above, but replaced the last parenthesis with ‘(marina*) NOT “*Zostera marina*”’. See Appendix S2 for further details of the search, including search string development, the number of publications found at each search stage and a list of database sub-files included in the search.

### Article screening and quality assessment

We screened the articles for inclusion in three successive steps. In the first step, a single reviewer screened the titles and abstracts for quantitative studies of boating activity impacts on submerged aquatic vegetation. Uncertain cases were rather included than rejected at this stage. In the next step, we read all articles that were included based on title and abstract. All the co-authors participated in reading and we discussed uncertain cases in the entire group. Studies were included if they passed each of the following criteria:

- Relevant subject population: Aquatic submerged soft-bottom vegetation, including vegetation in marine, brackish and freshwater environments.
- Relevant activity: Activity related to recreational boating or personal transport, including traffic and infrastructure for mooring (buoys and docks) of motorboats, sailboats, yachts, small-sized tourist ferries, smaller fishing boats and canal narrowboats and leisure barges up to 50 m in length.
- Relevant comparators: Areas or treatments with no or low occurrence of the activity.
- Relevant biological outcomes: Quantitative measures of total vegetation abundance, including biomass, cover or shoot density.
- Appropriate study design: Comparative studies, experimental field or mesocosm studies, or Before–After-Control-Impact (BACI) studies containing replicated data.

We excluded studies of anchor damage because most of these studies looked at recovery from small-scale physical damage and recovery in scars, rather than comparing areas or treatment with and without exposure.

Vegetation abundance was the only biological outcome that was investigated in a sufficient number of studies to allow a meaningful review and meta-analysis. Of the articles that appeared in our search, only nine investigated vegetation height (not more than two in any of the categories used in the analyses; Table 1), three growth rate, four species diversity and two species composition; we judged this too few to be included in the review.

**Table 1:** The four categories of human activities that were used in the analyses

Category	Description
Boat traffic	Studies comparing areas with different boat traffic intensity and experimental studies testing the effect of wake
Docks	Studies comparing vegetation below a dock with vegetation outside the effect zone of docks
Mooring buoys	Studies comparing vegetation within the reach of a buoy chain (swing zone) with vegetation outside the effect zone
Mooring areas	Studies comparing vegetation between water basins with mooring facilities (docks, jetties and buoy fields) for more than ten boats and water basins with no or very few mooring facilities

In the third step, all articles passing the described criteria were subject to critical appraisal, excluding studies and data where the methodology description was missing or with an inappropriate control. Studies with inappropriate control included comparisons where treatment and control were from different depth ranges, from different seasons or the “no boat-zone” lacked marker buoys (i.e. the designated area was only indicated as a recommendation on sign at the shore). We also excluded one control site with considerably higher vegetation abundance than the treatment site prior to impact by boating activity as well as one redundant article, with the same data as another article. A list of all articles that were excluded based on the full text assessment (step 2 and 3), and the reason for exclusion can be found in Appendix S3, together with a list of the articles that we failed to find in full text.

### Data extraction

Outcome means with measures of dispersion were extracted from text and tables, or from figures using the WebPlotDigitizer Software (Rohatgi 2016). In a few cases, summary statistics were calculated from data extracted from scatter plots. For two studies (Eriksson et al. 2004; Hansen and Snickars 2014), raw data was contributed by the authors to enable analysis at site level when the primary study reported averages across sites. In those cases, we calculated mean and variance per site and matched the impacted sites with reference sites to achieve comparisons between site pairs with similar geomorphological characters (depth, wave-exposure, water exchange).

Data from BACI-designs always included multiple readings after the intervention (e.g. the installation of a dock). We only used treatment data from the last sampling event to avoid bias created by subjective decisions of when the vegetation has reached equilibrium under the new condition. Further, to avoid dispersion created by variation between years or seasons we used the control from the same sampling event as the treatment. Thus, no data was used from before interventions from BACI-designs.

Beside the primary data, we recorded the following potential effect modifiers and metadata: species name, study system, name and coordinates of the study site, region, as well as study type (comparative, experiment or BACI) and if available, estimates of traffic intensity (e.g. the number of boat passages), number of berths, design of mooring infrastructure and water depth.

### Data handling

We synthesized the impact from boating activities on vegetation abundance through meta-analysis and meta-regression, together with a qualitative synthesis. For this purpose, we split the extracted data into four different categories based on the type of human activity that was investigated (Table 1). The first category comprised studies of the effects of boat traffic and artificially created wake. The second and third categories represented effects of infrastructure for mooring (docks and mooring buoys). The fourth category included studies comparing water basins with and without mooring facilities, representing the overall effect of mooring infrastructure and boat traffic on an entire water basin (hereafter “mooring areas”).

Estimates of traffic intensity were often absent or not comparable between the primary studies. However, for the mooring areas, we could use the number of berths (i.e. designated space where a single vessel may be moored on a jetty, pier, dock, buoy or alike) as a rough estimate of boating intensity. The number of berths or average number of moored boats was specified for all but one of the primary studies. For the remaining study, we counted the number of berths in a satellite image from the same year as the study was conducted, using Google Earth Pro (Version 7.1.5.1557) Historical Imagery. We further measured the size of boats in the mooring areas using Google Earth Pro historical satellite images (from the same years as the studies were conducted), if this was not specified in the primary studies. To enable calculation of the number of berths per hectare, we used the polygon-tool in the software Google Earth Pro to estimate the size of the area of the water basin utilized for mooring.

Dependent data were treated according to recommended procedures for meta-analyses (Borenstein et al. 2009; Koricheva et al. 2013). Such dependent data were for example multiple readings from the same investigated site, or more than one measurement of vegetation abundance in a single study. When single articles reported comparison of means from more than one site or survey (i.e. surveys conducted in the field at different occasions and/or in different sites), we included these as separate effect sizes in the analyses. Our reason to do this was that we expected more variation between sites than between studies. In addition, it enabled us to test the effect of the number of berths at site level; some of the articles included mooring areas with both high and low number of berths. We tested the impact of this decision on the outcome of meta-analyses with sensitivity analysis, which showed that the overall interpretation of the results was not affected by the choice of analysis level. Details on other types of dependent data are found in Appendix S4.

### Data synthesis and statistical analysis

We ran three separate meta-analyses to test for the effect of boat traffic, docks and mooring areas on vegetation abundance. We further explored the heterogeneity in effect sizes for mooring areas using meta-regression with the number of berths as co-variable. We tested both the number of berths per site and the number of berths per hectare of the site in separate meta-regression models. There was not enough data to test other potential co-variables.

We used log response ratios as effect size measure, ${\text{LRR}} = \ln (\bar{X}_{\text{I}} /\bar{X}_{\text{C}} ),$ where $\bar{X}_{\text{I}}$ corresponds to the mean vegetation abundance of the impacted treatment and $\bar{X}_{\text{C}}$ to the mean vegetation abundance of the control treatment (Borenstein et al. 2009). Negative LRR values thus reflect a lower vegetation abundance in the impacted treatment compared to the control treatment and positive values reflect the opposite. The summary effect was regarded to be significant if the 95% CI did not bracket zero. We regarded each set of effect sizes to be a range of estimated true effects rather than a range of estimates of a single true effect and used random-effect models allowing for variation between study effects in addition to sampling error. We used the DerSimonian Laird method to estimate variance between studies, applying inverse-variance weighting to account for variation in precision (sampling error) within and between studies. Calculations of effect sizes and all analyses were conducted with the software OpenMEE (Wallace et al. 2017). We assessed the potential impact of publication bias, e.g. lack of small studies with effect sizes close to zero or the influence of small studies with large effect size, on the summary effects (Appendix S5).

For the data of mooring buoys, we concluded that meta-analysis was unsuitable. For most of the buoy types explored, the dragging chain created a scour zone with complete or almost complete loss of vegetation close to the chain, and a gradual increase in vegetation abundance close to the fringe. Both the size of the scour zone and the placement of sampling differed between studies and buoy types, making comparison of effect size between studies irrelevant. Thus, we only present effects of mooring buoys qualitatively.

## Results

### General review statistics

**Fig. 2:**
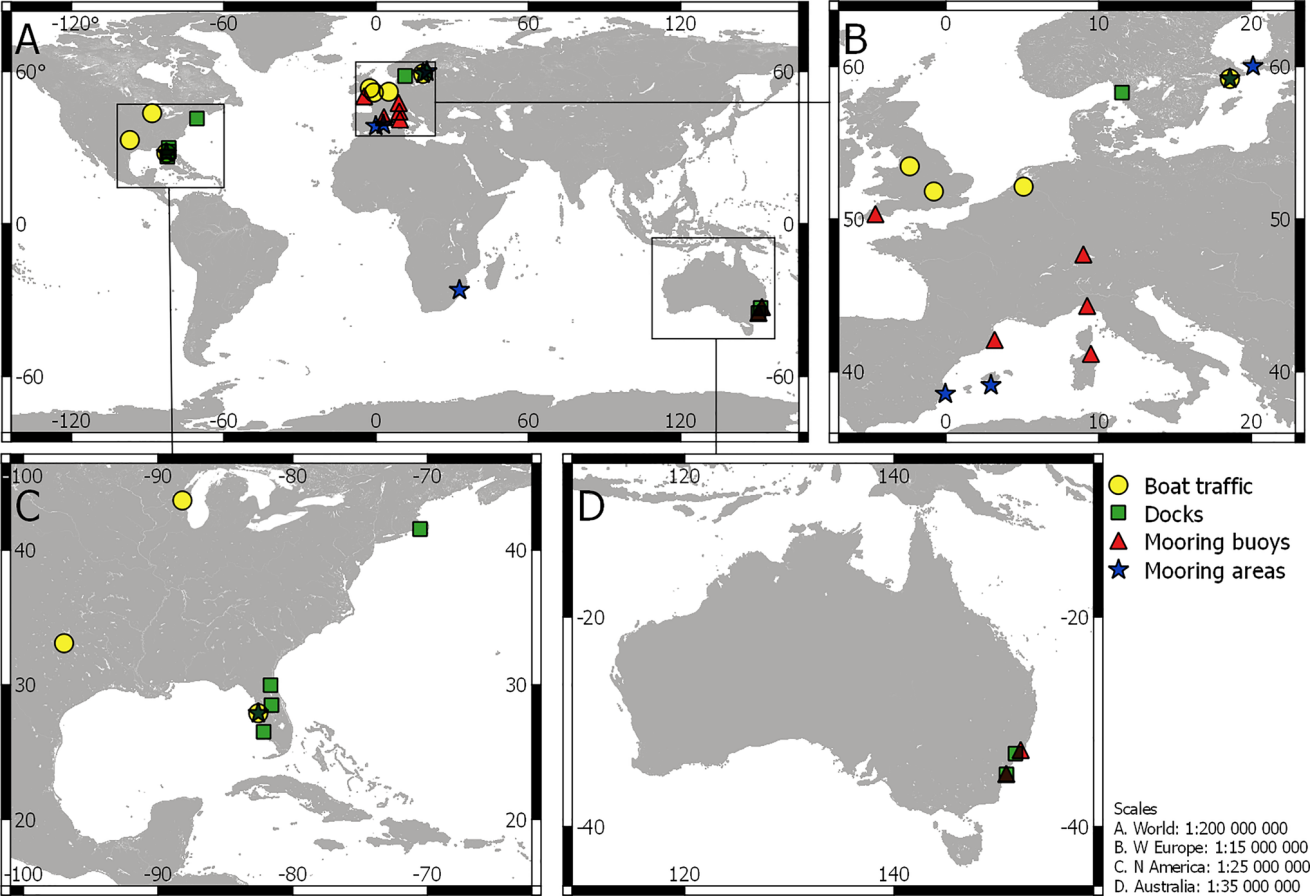
Geographical distribution of the studies that fitted the inclusion criterion and passed the critical appraisal, shown on **a** a world map with all studies, and separate regional maps for **b** Western Europe, **c** North America, and **d** Australia

The search resulted in 2499 unique hits, of which we reviewed 186 in full text. Of these, 25 articles fitted the inclusion criterion and passed the critical appraisal. The studies comprised both inland waters and coastal areas, but were almost exclusively conducted in temperate and subtropical areas in North America, Europe and Australia (Fig. 2). The majority were comparative studies. Seven articles included data on effects of boat traffic or artificially created wake, while data on docks and on mooring buoys occurred in seven, and data on mooring areas in six articles (Table S4). Two articles contributed with data to more than one data category. The impacts recorded were from several types of vessels, i.e. motorboats, sailboats, yachts, small-sized tourist ferries, smaller fishing boats, canal narrowboats and leisure barges.

### Boat traffic and wake

The seven articles contributing with data to this category were performed in a variety of systems, i.e. coastal areas (Eriksson et al. 2004; Mueller 2004), lakes (Asplund and Cook 1999; Doyle 2001), artificial canals (Murphy and Eaton 1983; Willby et al. 2001) and running waters (Vermaat and De Bruyne 1993) and studied the effects on either seagrass or mixed plant and algae communities (Table S4). Two articles presented experiments testing either the effect of artificially created wake (Doyle 2001) or the effect of breakwaters protecting from wake (Vermaat and De Bruyne 1993). The remaining articles presented comparisons of areas with different levels of traffic intensity. The type and size of the vessels trafficking these sites varied from small-sized tourist ferries (15–42 m) in Eriksson et al. (2004), pleasure boats and barges in the canal studies (reported to be 81% ≤ 9 m in one of the studies) to small recreational boats used for, e.g., fishing and water-skiing in Asplund and Cook (1999) (Table S4). We derived a total of 18 effect sizes for meta-analysis, where two-thirds originated from one study of coastal mixed plant and algae communities.

**Fig. 3:**
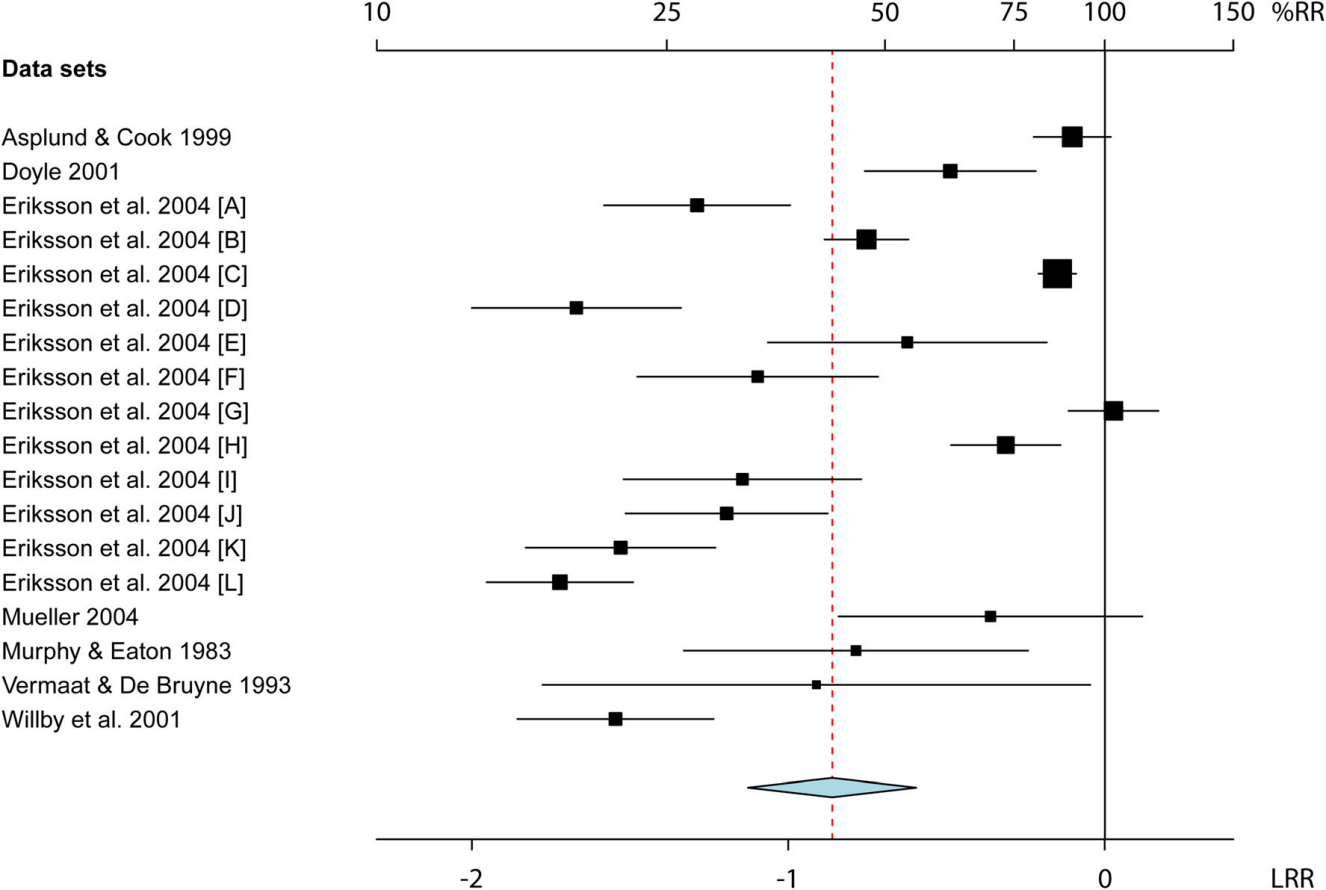
Mean effect size (log response ratio, LRR) of boat traffic within (black squares) and across (dashed line) studies or sites. The size of the black squares shows the weight of each data point in the analysis. Error bars and the diamond show 95% confidence intervals (CI_95_) for the means. The horizontal axis at the top of the figure shows the response ratio in percent (%RR), which is the equivalent of the average abundance in the impacted area compared to the control. The indexing (letters A–L) refers to different sites (see Table S5 for coordinates of the sites)

The vegetation abundance in areas with boat traffic and artificial wake was on average 42% of that in control areas (LRR = − 0.86 ± 0.27; mean ± 95% CI; *P* < 0.001; *N* = 18; Fig. 3). However, there was a significant heterogeneity (*Q*_T_ = 533.37; *P* (*χ*^2^) < 0.001; *I*^2^ = 96.81; *T*^2^ = 0.30), created by a large variation in effects between studies and sites. The effect size ranged between 18 and 100% abundance in sites or treatments with traffic or wake, compared to the abundance in controls, i.e. a very strong reduction to no effect. There was not enough data on potentially relevant co-variables (for instance traffic intensity, depth and habitat characteristics) to allow us to explore the cause of the heterogeneity through structured meta-analysis. The results were unlikely to be affected by publication bias or the way the data were aggregated (Appendix S5).

### Docks

The seven publications contributing with data to this category studied either coastal seagrass (Loflin 1995; Burdick and Short 1999; Fyfe and Davis 2007; Gladstone and Courtenay 2014; Eriander et al. 2017) or mixed freshwater vegetation (Steinmetz et al. 2004; Campbell and Baird 2009; Table S4). We derived 14 effect sizes for the meta-analysis; nine from comparative field studies, three from BACI surveys and one from an experiment (Steinmetz et al. 2004). One publication contributed with six of the data points. Distance to control areas varied between the studies, being located far from the docks (50 m to 5 km; Fyfe and Davis 2007; Gladstone and Courtenay 2014) or adjacent to the docks (Loflin 1995; Eriander et al. 2017). In the latter case, effects of boating on vegetation cannot be excluded as moored boats (Eriander et al. 2017) or traffic to and from the docks can affect the vegetation. Hence, these studies may underestimate the effect of docks.

**Fig. 4:**
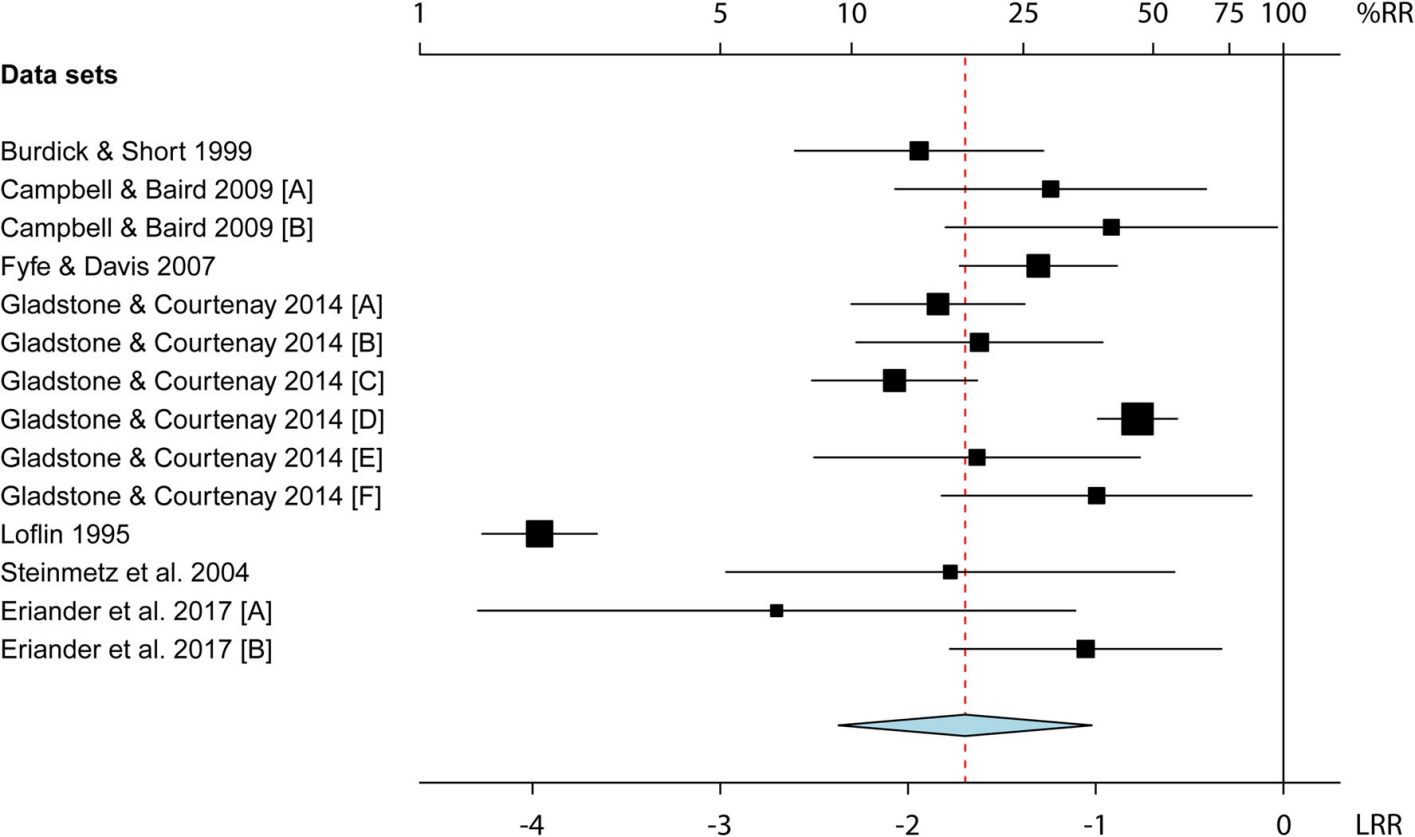
Mean effect size (LRR) of docks within (black squares) and across (dashed line) studies. The size of the black squares shows the weight of each point in the analysis. Error bars and the diamond show CI_95_ for the mean effect within and across studies. The horizontal axis at the top of the figure shows the response ratio in percent (%RR), which is the equivalent of the average abundance in the impacted area compared to the control. For Campbell and Baird (2009), the indexing (letters A, B) refers to different sites (see Table S5 for coordinates of the sites). For Gladstone and Courtenay (2014), it refers to comparative studies of docks with cardinal direction E–W (A) or N–S (B), of docks with decking of wood (C) or mesh (D), and to BACI studies of wood (E) or mesh (F) docks. For Eriander et al. (2017), it refers to studies of floating (A) and fixed (B) docks, respectively

The vegetation abundance underneath docks was on average 18% of that in control areas (LRR = − 1.70 ± 0.67; mean ± 95% CI; *P* < 0.001; *N* = 14; Fig. 4). However, there was a significant heterogeneity (*Q*_T_ = 302.80; *P* (*χ*^2^) < 0.001; *I*^2^ = 95.71; *T*^2^ = 1.50). The vegetation abundance underneath docks was 9–36% of that in the control areas. There was large variation in the reporting and testing of factors that could modify the effect of docks on vegetation, such as dock design and water depth (Table S4). This, together with the small number of studies, meant that we could not explore the reasons for heterogeneity through structured meta-analysis.

The results were unlikely to be affected by publication bias or the way the data were aggregated (Appendix S5). Loflin (1995), however, appeared as an outlier in the sensitivity analysis. When excluding the data point from the analysis the summary effect decreased in magnitude from − 1.70 to − 1.47 (i.e. a change from 18 to 23% cover underneath docks compared to controls). However, the overall interpretation of the result is not affected by the presence or absence of this data point (Appendix S5).

### Mooring buoys

Six of the articles reporting effects from mooring buoys were performed in coastal seagrass meadows (Montefalcone et al. 2006; Demers et al. 2013; La Manna et al. 2015; Colomer et al. 2017; Unsworth et al. 2017; Glasby and West 2018) and the remaining one in a charophyte-dominated vegetation community in a lake (Ostendorp et al. 2009). Five different mooring types were studied. Conventional “swing moorings”, with a chain moving along the bottom around a central anchor, were included in six of the studies. The data also included three mooring types constructed to reduce scouring of benthic organisms; a “hook buoy” where the length of the chain is adjusted to reduce bottom contact; a “cyclone mooring” with a system of three chains extending in different directions on the seabed; and a “screw mooring” with a small moving rod that is elevated from the bottom to which the buoy rope is connected. Finally, one study looked at the effect of a chain system running along the bottom between anchor points. Only the swing mooring was studied multiple times.

The majority of the mooring types had a marked impact on the vegetation in their immediate surrounding, often reducing the vegetation with 100% close to the chain. Only the areas with “screw mooring”, where the chain is not in direct contact with the bottom, had a similar cover to the nearby reference areas. Since the vegetation abundance data were collected at different distances from the buoy in the different studies, we did not test for a common main effect of buoys through meta-analysis.

The reported size of the scarred area (with no or very low cover of vegetation) created by a single buoy varied between a few to over 1000 m^2^, depending on buoy construction. For swing moorings, the size of the scar depended on the length of the bottom chain (e.g. Ostendorp et al. 2009; Glasby and West 2018). Consequently, the “hook buoy” that allowed adjustment of the chain length to the water level, strongly decreased the scar size (from an average of 87 to 6 m^2^) in a freshwater system with fluctuating water level (Ostendorp et al. 2009). The most extensive scars were created by the “cyclone mooring” with three long chains (Demers et al. 2013).

### Mooring areas

Six articles contributed with data on mooring areas from comparative field studies in coastal habitats (Marbà et al. 2002; Eriksson et al. 2004; Mueller 2004; Fernandez-Torquemada et al. 2005; Nordlund and Gullström 2013; Hansen and Snickars 2014). We derived 25 effect sizes for meta-analysis, where each point consisted of one mooring area compared to an individual reference area. The majority of the data were from two studies of mixed plant and algae communities in the brackish Baltic Sea (Eriksson et al. 2004; Hansen and Snickars 2014). The rest of the studies were from subtropical seagrass. The data covered a large range of mooring intensity, from a few berths to several hundred per site. The size of boats in these mooring areas were predominantly ≤ 15 m in length (96%), but a few boats were > 15 ≤ 25 m (4%) and only exceptionally the boats were > 25 ≤ 50 m (< 1%).

**Fig. 5:**
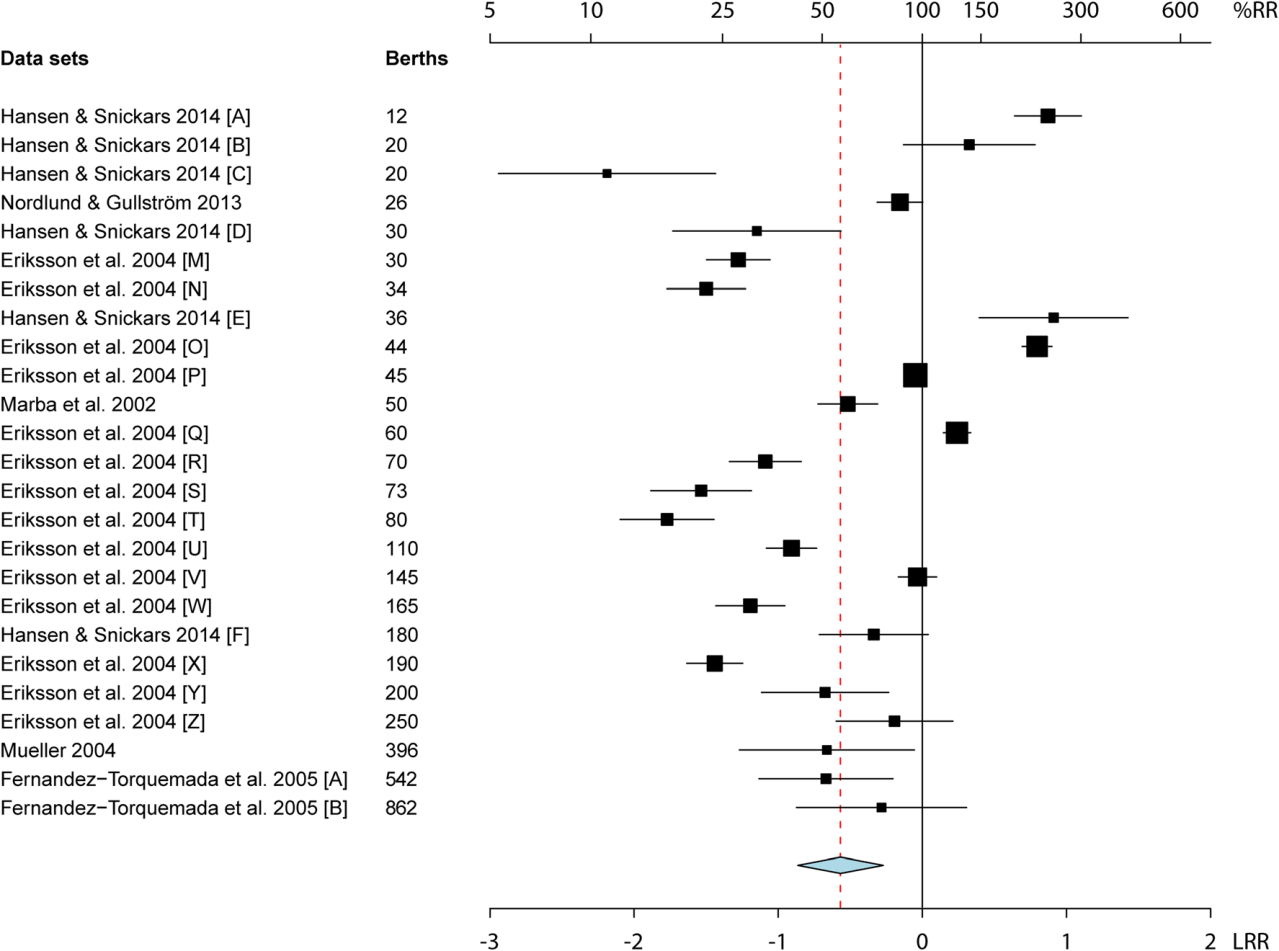
Mean effect size (LRR) of mooring areas within (black squares) and across (dashed line) sites. The sites are sorted from lowest to highest density of berths per site. The size of the squares shows the weight of each data point in the analysis. Error bars and the diamond show CI_95_ for the mean effect within and across sites. The horizontal axis at the top of the figure shows the response ratio in percent (%RR), which is the equivalent of the average abundance in the impacted area compared to the control. The indexing (letters A–Z) refers to different sites (see Table S5 for coordinates of the sites)

Meta-analysis showed an average effect size of 57% vegetation abundance in mooring areas compared to control areas (LRR = − 0.57 ± 0.30; mean ± 95% CI; *P* < 0.001; *N* = 25; Fig. 5). However, there was a significant heterogeneity due to large variation in effect between sites (*Q*_T_ = 1279.06; *P* (*χ*^2^) < 0.001; *I*^2^ = 98.12; *T*^2^ = 0.54) and there was a slight risk of publication bias with the smallest studies inflating the summary effect (Appendix S5). Hence, the mean summary effect should be viewed as indicative and variation in effects sizes should be explored. The effect size of the analysed data sets varied between 11 and almost 250% abundance in mooring areas compared to control areas. Neither of the two meta-regression models showed a significant effect of mooring intensity on vegetation abundance (*P* = 0.93 and 0.69 for total number of berths per se and berths per ha, respectively; Appendix S7). However, we noted that all sites with an average positive effect size had a relatively low density of berths per site (in the lower two quartiles of the data).

## Discussion

Our review showed that recreational boat traffic and infrastructure for mooring can have a significant impact on the abundance of submerged aquatic vegetation in freshwater and coastal systems. Vegetation abundance in areas or experimental treatments exposed to boat traffic was on average of 42% of the abundance in control areas or treatments, but varied between 18 and 100%. Thus, while recreational boating rarely leads to large-scale depletion of submerged vegetation, our results suggest that it is likely to cause significant decreases in vegetation abundance. Such thinning has potential to affect the ecological functions provided by the vegetation. For instance, several studies indicate that the ability of submerged vegetation to reduce turbidity is related to the abundance or areal extent of vegetation (Orth et al. 1999; Moore 2004; Austin et al. 2017). Moreover, the abundance of macroinvertebrates (Diehl and Kornijów 1998; Attrill et al. 2000), as well as juvenile fish (Hansen et al. 2018; Kraufvelin et al. 2018), has been shown to increase with vegetation abundance. Hence, vegetation decline caused by boating activities may substantially degrade the habitat quality provided by vegetation in the otherwise flat seascape of soft bottoms.

Construction of docks over vegetated habitats always resulted in more than 50% reduction in vegetation abundance and on average the abundance below docks was only 18% of that in control areas. We could not calculate a comparable mean effect for mooring buoys, but most of the investigated mooring types resulted in a complete or almost complete loss of vegetation in the reach of the buoy chain. The effect was restricted to the area below or in direct vicinity of the dock or buoy, which means that a single dock or buoy affected a relatively small area. Still, in regions with high recreational boat density the cumulative effect of mooring infrastructure can be considerable (e.g. Hastings et al. 1995; Eriander et al. 2017; Glasby and West 2018), in particular since docks and buoys often are placed in areas that are sheltered from winds and waves—the most suitable areas for submerged vegetation. Moreover, when mooring buoys cause fragmentation of continuous seagrass meadows in coastal areas it increases the risk for further seagrass loss through erosion (Hastings et al. 1995).

In mooring areas, which represent the overall effect of mooring infrastructure and boat traffic on an entire water basin, the vegetation abundance was in many cases lower, but sometimes higher, than in control areas. This indicates that the effect of docks and buoys may extend outside the immediate effect zone, possibly by increased boat traffic around the mooring facilities. However, the variation was large between studies and sites, and more research is needed to show when and where the construction of mooring facilities is likely to affect vegetation over a larger area.

### Why do the effects vary?

All the meta-analyses showed significant heterogeneity, demonstrating that the effect of docks, traffic and mooring areas varied across studies and sites. Our dataset was not large enough to allow us to conclude on reason for this variability, but we discuss the available evidence from the scientific literature below. The analyses included sites with widely different vegetation and environmental characteristics, which are likely to differ in sensitivity to disturbance from recreational boats. For instance, the bottom type influences how easily water turbulence from motors and wake stir up the bottom sediment that increases turbidity and decreases availability of light for the vegetation (Fig. 1). Fine silt and organic particles are easier to stir up and stays longer in the water column compared to sand and coarser sediment. Since such easily suspended matter typically accumulates in naturally wave-sheltered areas, such as small ponds, creeks and enclosed bays, these habitats may be particularly sensitive to boat-induced wake and turbulence (e.g. Klein 1997).

The water depth is also likely to affect the sensitivity of submerged vegetation habitats to boating disturbance. Sediment resuspension, as well as direct damage to the vegetation from water turbulence and propellers, occur primarily in shallow areas. Several studies have found that significant sediment resuspension arise when boats are operating in waters less than around 2.5 m deep (Yousef 1974; Gucinski 1982; Klein 1997). Similarly, scarring from boat propellers in seagrass beds occurs mainly in areas less than two meters deep (Sargent et al. 1995). On the other hand, an increased turbidity or shading from dock constructions can have the largest effect in deeper areas, where light availability is the main factor limiting vegetation growth (Krause-Jensen et al. 2008). Accordingly, Eriksson et al. (2004) showed that vegetation abundance declined more rapidly with depth in areas exposed to boat traffic, compared to control areas. Low light levels in deep areas may also slow down recovery from disturbance (Montefalcone et al. 2006).

Different vegetation species are likely to differ in sensitivity to disturbance from boating. For instance, fast-growing species that can elongate and concentrate much of their photoreceptive biomass near the surface are more capable than small slow-growing species to compensate for low light conditions in turbid conditions (Barko and Smart 1981; Boston et al. 1989; Duarte and Roff 1991; Hansen and Snickars 2014). Similarly, non-attached free-living species are more tolerant to reduced light conditions, as well as bottom disturbance, since they live close to the surface. Accordingly, in mixed species communities the composition has shifted to a dominance of such species tolerating high turbidity in response to boating disturbance (Murphy and Eaton 1983; Asplund and Cook 1999; Willby et al. 2001; Eriksson et al. 2004; Hansen and Snickars 2014). Growing on or close to the water surface may, however, increase the exposure for physical damage by propellers, hulls and wake (Murphy and Eaton 1983).

In addition to differences in sensitivity to boating disturbance between species and ecosystems, the effect of boat traffic is likely to be higher when the traffic is intense than when only one or a few boats are moving in an area. This is supported by the negative relationship between traffic intensity and vegetation abundance that was documented in the two primary studies that included a range of boating intensities (Murphy and Eaton 1983; Willby et al. 2001). Both studies were performed in narrow and shallow man-made canals, where the number of lockage operations provided a good estimate of boating intensity in different canal sections.

In contrast, we did not find any relationship between the number or density of berths in mooring areas and the impact on vegetation abundance. We could see several possible reasons for this. For instance, as discussed above, the sensitivity to boating disturbance is likely to differ between species and habitats, obscuring any general relationship between boating intensity and effect across ecosystems. Also, the number of berths may be a crude measure of boating intensity. However, a recent study by Hansen et al. (2018) found that the abundance of rooted vegetation decreased significantly with the density of berths in Baltic Sea coastal bays, indicating that such relationship may exist—at least for rooted species.

When it comes to docks, the effect on submerged vegetation depends on how they are designed and placed. Although we could not evaluate the effect of certain designs across studies, one overall conclusion is that designs that reduce the shading effect of the dock to some extent can reduce the negative impact on vegetation below the dock. Docks can reduce the photosynthetic active radiation at the seabed to < 10% of the level at comparable depth (Steinmetz et al. 2004; Campbell and Baird 2009). Since growth and survival of submerged vegetation depends on the incoming light, it is likely that the permanent shade created by docks is the major factor causing vegetation to decline under and adjacent to the dock, although other factors may contribute (e.g. direct or indirect disturbance from boats operating near the dock). The strongest shading is created by floating docks, which have no space between the deck and the water surface. Accordingly, comparisons between floating docks and docks that are elevated over the surface have shown that the former have a more negative impact on vegetation below the dock (Burdick and Short 1999; Eriander et al. 2017; Fig. 4). Shading may also be reduced by using decking materials that allow light penetration (e.g. aluminium mesh; Gladstone and Courtenay 2014) and by orienting the dock in north–south direction so that the sunlight can reach the bottom under the dock during most of the day (Burdick and Short 1999; Campbell and Baird 2009; Fig. 4).

**Fig. 6:**
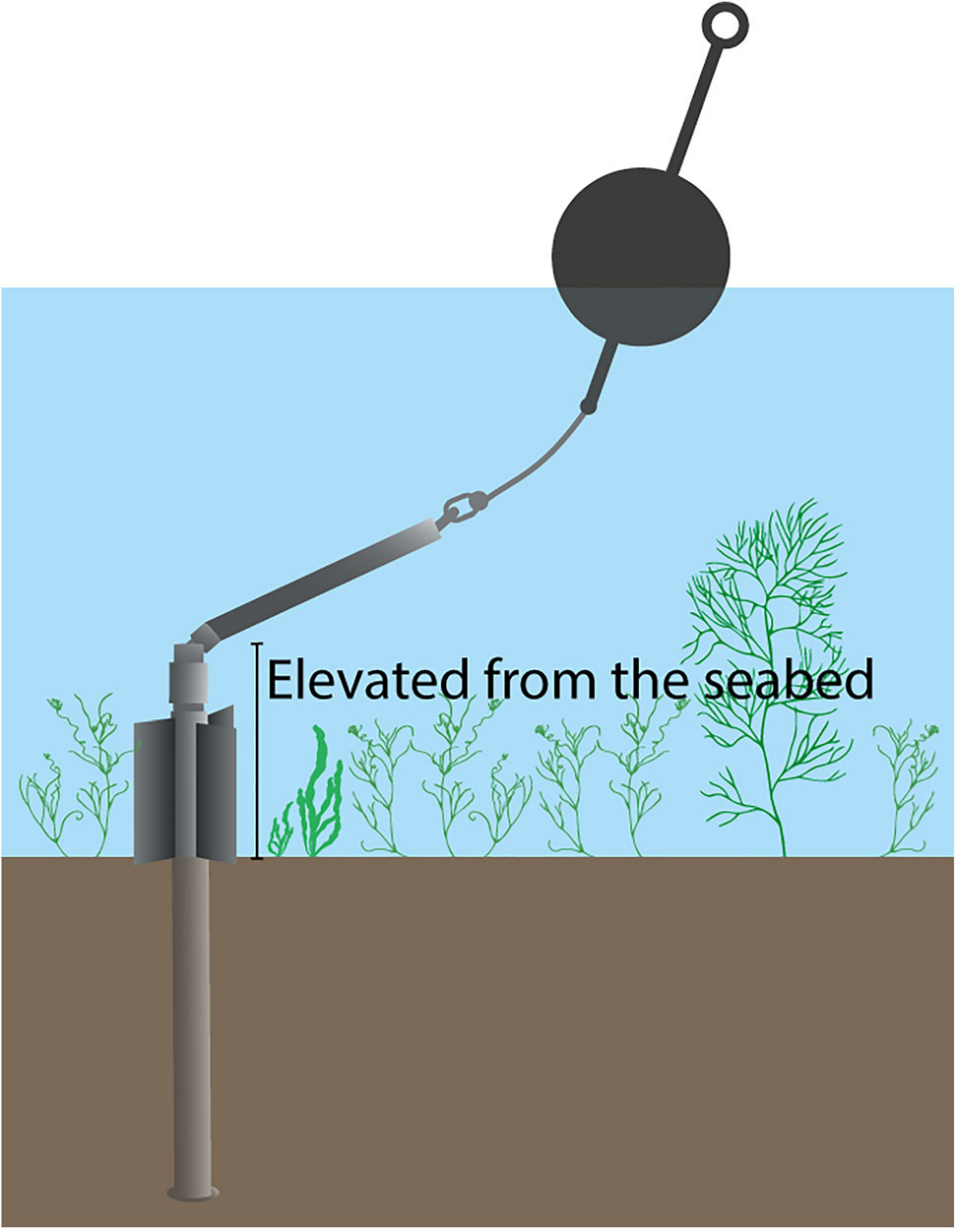
Illustration of a mooring buoy that was found to not cause any significant loss of vegetation in one of the included studies. Redrawn from Demers et al. (2013)

Mooring buoys mainly affect the vegetation through physical damage from chains dragging over the bottom. This means that the damage from a single buoy can be reduced by decreasing the bottom area that is exposed to moving chains. For instance, Ostendorp et al. (2009) showed that conventional swing moorings caused loss of vegetation cover over a 15 times larger bottom area than buoys with adjustable chain length. The only mooring type in our data that was designed to move without a dragging chain (“screw mooring” with a moving rod elevated from seabed; Fig. 6) did not cause any significant loss of vegetation cover (Demers et al. 2013).

### Implications for research

We identified a number of research gaps that hindered us from answering our initial research questions. Firstly, there is a general need for more quantitative studies of the effects of recreational boat traffic and mooring on submerged vegetation. The low number of studies precluded us from evaluating what factors are important to explain the heterogeneity in effect size between studies and sites. For instance, more studies would allow testing which habitats and species are the most sensitive to boating disturbance. One testable hypothesis is that functional traits, such as size, growth rate and regenerative capacity, affect species´ sensitivity to boating disturbance. Another is that species-rich communities are more resilient to disturbance due to complementary species traits that buffer against negative effects on the total vegetation community abundance. Understanding the most important factors affecting vegetation sensitivity would allow us to better predict the effect in a certain area and possibly steer away intensive traffic from the most sensitive habitats.

Secondly, more studies measuring the response of vegetation over a gradient of traffic intensities would allow a better description of the relationship between the pressure and vegetation abundance, for instance if there are threshold levels of traffic over which the vegetation in a specific habitat will be strongly affected. Such non-linear relationship was seen in the primary studies that tested for the effect of traffic intensity on vegetation abundance in artificial canals (Murphy and Eaton 1983; Willby et al. 2001). Further, better measurement and standardized reporting of the amount and type of boat traffic will allow future meta-analyses to test for effects of intensity across studies.

Thirdly, it was only for the response of vegetation abundance that we found a sufficient number of studies to allow a meaningful synthesis. If species differ in sensitivity to boating disturbance, measuring only total abundance could hide substantial change in vegetation communities with potential implications for biodiversity and ecosystem function. More studies of effects on species diversity and composition of the submerged vegetation are needed to complete the picture of boating effect on vegetation communities. In addition, studies investigating the effects on vegetation-associated species such as fish, invertebrates, birds and mammals would help evaluate the broader ecological effects of the documented changes in vegetation.

### Implications for management

The effect of boat traffic on submerged vegetation has been studied quantitatively in a wide range of marine and freshwater systems, but the number of studies is still too small to give general management recommendations across systems. On the one hand, the meta-analysis revealed that the average effect of boat traffic was more than 50% loss in vegetation abundance, showing that recreational boat traffic can be considered a significant pressure to submerged vegetation. On the other hand, the clearest result was that the effect was very variable and the evidence base is too scattered to allow clear advice on when and where to expect a large effect.

A few simple rules of thumb can be deduced from our current understanding of boat traffic impacts. For instance, the effect of boat-induced wake and turbulence is likely to have the largest effect in environments that are naturally wave-sheltered with easily stirred fine sediment bottoms, such as small ponds, lakes and creeks and enclosed bays. Direct negative effects from propeller scarring and hull groundings are mainly restricted to very shallow areas, less than 2.5 m deep. Beyond these simple guidelines, the only way to assess the risks for disturbance from boat traffic in a certain area is to measure its actual impact on vegetation. We call for a further collaboration between management and research, where eventual management interventions to restrict or permit boat traffic are well monitored. This will allow refinement of management over time and development of better future guidelines for management of recreational boat traffic in shallow coastal and inland water areas.

While the effect of boat traffic is variable, the existing evidence shows that construction of docks and mooring buoys in vegetated habitats in most cases lead to loss of vegetation or a strong reduction in vegetation abundance below the dock or in the reach of the mooring chains. This shows that it is important to consider the potential effects on submerged vegetation, including cumulative effects, when constructing docks and buoys for mooring of recreational boats. In popular boating sites, construction of mooring buoys may still be a strategy to relieve sensitive vegetation from anchoring damage, in particular if the buoys are constructed to diminish the damage to the vegetation (Fig. 6). Also for docks, choosing the best possible design that diminish the shading of the bottom (e.g. elevated instead of floating docks; Burdick and Short 1999; Eriander et al. 2017) can reduce the negative effect to some extent.

Apart from the direct shading and scour from docks and moorings, increased boat traffic around the mooring sites can lead to decreased vegetation abundance in the surrounding area (e.g. Burdick and Short 1999; Eriander et al. 2017). Such indirect effects are important to consider when deciding where to locate infrastructure for boat mooring. For instance, the common practice to place mooring infrastructure in wave-sheltered bays may put a disproportionate pressure on a sensitive system that constitute important habitats both for the vegetation and associated fauna (e.g. Sundblad and Bergström 2014).

## Conclusion

Despite the limited number of studies that fitted the review criteria, three important conclusions emerge from our review. Firstly, both recreational boat traffic and infrastructure for mooring (docks and buoys) can have a significant impact on the abundance of submerged aquatic vegetation in freshwater and coastal systems, which need to be considered in management. Secondly, the effect of traffic and mooring infrastructure is variable and range from no reduction in abundance to a complete loss of vegetation. This suggest that the impact can be reduced by restricting boating activities in areas with high risk for negative effects. Thirdly, to move us beyond the general conclusion that recreational boating *can* have an impact on vegetation habitats, there is a need for more quantitative studies. More studies from different systems would allow us to predict when submerged vegetation is at risk from boating activities and to reduce the impact, which is critical for balancing the benefits from recreational activities against nature protection.

## Electronic supplementary material

Below is the link to the electronic supplementary material.

Supplementary material 1 (PDF 733 kb)
