# Supplementary material for: Effects of boat traffic and mooring infrastructure on aquatic vegetation: A systematic review and meta-analysis
Source: Ambio. 2019 Jul 11;49(2):517–30. doi: 10.1007/s13280-019-01215-9 (PMC6965043; doi:10.1007/s13280-019-01215-9)
Supplement: Supplementary file 1 — Supplementary material 1 (PDF 733 kb) [file 13280_2019_1215_MOESM1_ESM.pdf]

Ambio

Electronic Supplementary Material

Title: **Effects of boat traffic and mooring infrastructure on aquatic vegetation: A systematic review and meta-analysis**

Authors: J Sagerman, J P Hansen, S A Wikström

https://doi.org/10.1007/s13280-019-01215-9

Appendix S1. Review protocol

Background

Submerged plant communities constitute a crucial part of the near shore ecosystems in coastal areas, lakes and rivers. They offer food and shelter to invertebrates and fish, and provide several important ecosystem functions and services, such as, primary production, carbon binding, sediment stabilization and filtration of land run off (Hemminga and Duarte 2000; Madsen et al. 2001; Fourqurean et al. 2003; Scheffer 2004; McGlathery et al. 2007). Human activities are intense in near shore areas, and the aquatic plant communities are under pressure from a number of stressors, such as, nutrient input from sewage and agriculture, fishing, the construction and use of marinas and intense boat traffic (Klein 1997; Newell et al. 1998; Howarth et al. 2000; Hughes et al. 2004). These stressors may affect the aquatic environment in different ways that are associated with negative impact on biota, for instance, through reducing the light reaching submerged plant communities (Klein 1997; Eriksson et al. 2004; Hughes et al. 2004). Coastal areas, rivers and lake systems have lost a large amount of vegetation the last century (Scheffer 2004; Lotze et al. 2006; Waycott et al. 2009). To ensure a future coexistence between well-functioning ecosystems and high recreational values in near-shore areas, the pressure from human activities, including recreational use, needs to be evaluated and managed.

Recreational boating is an activity that has increased strongly since the 1960s (Aall et al. 2011; Burgin and Hardiman 2011; Sundblad and Bergström 2014). It holds great value to society through providing recreational opportunities for many people and may contribute substantially to local and regional economy (Hassan et al. 2005; Ghermandi and Nunes 2013). However, as with many human activities, recreational boating may come with an environmental cost. The environmental stressors associated with recreational boating can be of physical, chemical or biological character, e.g., altered hydrodynamic forcing and resuspension of sediment by boat mediated wake, leaking antifouling paint and transportation of non-native species (Mosisch and Arthington 1998). For the purpose of this review, we will focus on physical stressors from boat traffic, anchoring and infrastructure for mooring (i.e., docks and mooring buoys) that may or may not be aggregated in “mooring areas” (i.e., marinas, harbors and buoy fields, intended for recreational boats).

Existing research suggests that the impact of boating activities depends on:

- The depth at the site
- The intensity of boating activity
- The size, type and speed of the boat
- The type of mooring facility (type of dock, jetty or bouy)
- The habitat and macrophyte community/ species subjected to disturbance
- The sediment grain size in the habitat
- The level of natural disturbance that occurs at the site (e.g. wind fetch, seasonal variation)

The aim of this project is to assemble a qualitative and a quantitative synthesis of the physical impacts of recreational boating on submerged aquatic vegetation, using a systematic review approach. We will summarize the current knowledge on the impact of boat traffic, infrastructure for mooring (docks and mooring buoys), as well as mooring areas (i.e., marinas, harbors and buoy fields, intended for recreational boats), to answer one primary and one secondary question.

Primary question:

How large is the magnitude of the effects from recreational boating on submerged aquatic vegetation growing on soft substrate?

Secondary question:

Does the effect of recreational boating differ in magnitude depending on boating intensity, depth, biogeographical region, type of environment or vegetation type?

Methods

The methods will be designed to identify and summarize, evidence from experimental studies (e.g. before/after, control/impact) and comparative studies (spanning a gradient of recreational boating intensity) to identify changes in e.g. abundance, biomass, species diversity and species composition of submerged aquatic vegetation (angiosperms and seaweeds) growing on soft substrates.

Search strategy

A search query will be constructed using Boolean operators (e.g., AND/OR/NOT) and “wildcards” (i.e., symbols that can be substituted for any character to enable search hits with different spelling or word endings). The specific search terms will be derived from the primary research question, using descriptors and similes of the four “PICO components”; population, intervention, comparator and outcome (Table S1). The search query will be developed sequentially, using ISI Web of Science, to balance the sensitivity against the specificity of the search. The quality of the search will be continually monitored through checking the titles of the search hits. The finalized search query will be used in the online citation databases; the ISI Web of Science and ASFA; Aquatic Science and Fisheries Abstracts, with subdirectories. The aim will be set to identify relevant studies published in both reports and peer-reviewed journals. The search terms will be confined to English, but search hits in other languages will be allowed, and translated for evaluation. All search hits retrieved from the final search will be imported to a reference handling software (Zotero), to facilitate deletion of duplicates and evaluation of relevance.

Article screening

The studies will be evaluated for inclusion/ exclusion in three successive steps. At the first step, the titles and abstracts will be screened for studies containing empirical data of physical impacts on submerged aquatic vegetation from recreational boating. Uncertain cases will be retained for step two. At the second step, the articles will be read at full text. At the full text level all studies that fits the five criteria specified in Table S1 and contains quantifiable, replicated measure of the response will be retained for the last step. At the third and last step, the studies will be subjected to critical appraisal. Studies with seriously defective methodology will be excluded at this stage. Studies that have been excluded at the second and third step of the screening will be listed together with reasons for exclusion and published as supplementary material along with the manuscript.

Table S1 Definitions of components of the primary review question

**Subject population**	**Intervention**	**Comparators**	**Biological outcome measures**	**Designs**
Aquatic submerged soft-bottom vegetation in coastal and inland waters	Exposure related to recreational boating, i.e., traffic, docks, jetties, piers, mooring buoys, marinas and other mooring areas	Areas/ treatments with no or very low exposure to recreational boating	Vegetation abundance (cover, biomass and shoot density) and other relevant measures of vegetation communities	Experiments, comparative studies and ‘Before & After, Control & Impact’ studies (BACI)

Data extraction and synthesis

The studies that pass the article screening will be summarized in a qualitative synthesis, where ecological and methodological key traits will be highlighted to enable comparisons. Initial literature scooping indicates that there are sufficient amounts of data for quantitative synthesis. Data will be extracted from tables, figures and text to enable calculation of effect sizes to answer our primary question. Potential effect modifiers and metadata will also be recorded (i.e. details specified in the bulleted list above and in Table S1). Standardized meta-analytical methods will be used for the purpose of quantitative analysis. Before analysis, the data will be divided into different categories based on the type of intervention and outcome measure. Analysis will be conducted where sufficient amount of data is present (i.e., data from at least 4–5 independent studies). The between study variation will be estimated and when possible, the influence of potential effect modifiers will be explored in meta-regression or subgroup analysis. Possible presence and influence of publication bias will be explored with standardized methods. Sensitivity analysis will be conducted to explore the impact of subjective decisions on the summary effects.

Substantive amendments of the protocol

Studies relating to anchoring damage were omitted 2017-09-07, because the majority of the studies focused on vegetation recovery from small-scale damage, while the studies of other exposure categories (e.g. boat traffic and mooring facilities) compared areas and treatments with and without exposure. The ability of the vegetation to recover is an important aspect that affects the impact of recreational boating, but time and resource constrains made us exclude the studies of anchor damage.

Data related to the biological outcome measures of vegetation height, growth rate, species diversity and species composition were omitted 2017-10-02, because there were too few studies turning up in our literature search (not more than two studies for any type of exposure).

References

Aall, C., I. G. Klepp, A. B. Engeset, S. E. Skuland, and E. Støa. 2011. Leisure and sustainable development in Norway: part of the solution and the problem. *Leisure Studies* 30: 453–476.

Begg, C. B., and M. Mazumdar. 1994. Operating characteristics of a rank correlation test for publication bias. *Biometrics*: 1088–1101.

Borenstein, M., L. V. Hedges, J. Higgins, and H. R. Rothstein. 2009. *Introduction to Meta-Analysis*. West Sussex, UK: Wiley.

Burgin, S., and N. Hardiman. 2011. The direct physical, chemical and biotic impacts on Australian coastal waters due to recreational boating. *Biodiversity and Conservation* 20: 683–701.

Eriksson, B. K., A. Sandström, M. Isaeus, H. Schreiber, and P. Karås. 2004. Effects of boating activities on aquatic vegetation in the Stockholm archipelago, Baltic Sea. *Estuarine, Coastal and Shelf Science* 61: 339–349.

Fourqurean, J. W., L. M. Rutten, M. Durako, J. C. Zieman, and T. A. Frankovich. 2003. Assessing Change in Seagrass Ecosystems on a Regional Scale: The Relative Merits of Fixed Stations and Systematic Random Sampling. *Gulf of Mexico Science* 21: 133.

Ghermandi, A., and P. A. L. D. Nunes. 2013. A global map of coastal recreation values: Results from a spatially explicit meta-analysis. *Sustainable Urbanisation: A resilient future* 86: 1–15.

Hassan, R., R. Scholes, and N. Ash. 2005. *Ecosystems and human well-being: current state and trends, vol 1. Findings of the condition and trends working group of the Millennium Ecosystem Assessment*. Washington, DC: Island Press.

Hemminga, M. A., and C. M. Duarte. 2000. *Seagrass ecology*. Cambridge University Press.

Howarth, R. W., D. B. Anderson, J. E. Cloern, C. Elfring, C. S. Hopkinson, B. Lapointe, T. J. Maloney, N. Marcus, et al. 2000. Issues in ecology: Nutrient pollution of coastal rivers, bays, and seas.

Hughes, A. R., K. J. Bando, L. F. Rodriguez, and S. L. Williams. 2004. Relative effects of grazers and nutrients on seagrasses: a meta-analysis approach. *Marine Ecology Progress Series* 282: 87–99.

Klein, R. 1997. *The effects of marinas and boating activity upon tidal waterways*. Owings Mills, Maryland: Community & Environmental Defense Services.

Lotze, H. K., H. S. Lenihan, B. J. Bourque, R. H. Bradbury, R. G. Cooke, M. C. Kay, S. M. Kidwell, M. X. Kirby, et al. 2006. Depletion, degradation, and recovery potential of estuaries and coastal seas. *Science* 312: 1806–1809.

Madsen, J. D., P. A. Chambers, W. F. James, E. W. Koch, and D. F. Westlake. 2001. The interaction between water movement, sediment dynamics and submersed macrophytes. *Hydrobiologia* 444: 71–84.

McGlathery, K. J., K. Sundbäck, and I. C. Anderson. 2007. Eutrophication in shallow coastal bays and lagoons: the role of plants in the coastal filter. *Marine Ecology Progress Series* 348: 1–18.

Mosisch, T. D., and A. H. Arthington. 1998. The impacts of power boating and water skiing on lakes and reservoirs - A review. *Management* 3: 1–17.

Newell, R. C., L. J. Seiderer, and D. R. Hitchcock. 1998. The impact of dredging works in coastal waters: A review of the sensitivity to disturbance and subsequent recovery of biological resources on the sea bed. *Oceanography and Marine Biology* 36: 127–178.

Rosenthal, R. 1979. The file drawer problem and tolerance for null results. *Psychological bulletin* 86: 638.

Scheffer, M. 2004. *Ecology of shallow lakes*. Dordrecht: Springer Netherlands.

Shenhav, L., R. Heller, and Y. Benjamini. 2015. *Quantifying replicability in systematic reviews: the r-value*. Tecnical report arxiv1502.00088. Technical Report Arxiv1502.00088. Tel-Aviv: Tel-Aviv University.

Sundblad, G., and U. Bergström. 2014. Shoreline development and degradation of coastal fish reproduction habitats. *Ambio* 43: 1020–1028.

Waycott, M., C. M. Duarte, T. J. B. Carruthers, R. J. Orth, W. C. Dennison, S. Olyarnik, A. Calladine, J. W. Fourqurean, et al. 2009. Accelerating loss of seagrasses across the globe threatens coastal ecosystems. *Proceedings of the National Academy of Sciences* 106: 12377–12381.

Appendix S2. Report of literature scoping

Defining the search terms

We used the PICO approach to identify the four components; population, intervention, comparator and outcome, in our research question and based the search string development on the first three components. The search string was developed sequentially, by adding one search term at a time and evaluating the number of additional hits and the relevance of the returned titles. The search was performed using ISI Web of Science with the time span set to all years (1945–2015). The tested search terms for population included the genus or phylum name of the most common vegetation on soft bottoms.

The following search terms were retained since they returned at least one relevant article when added to the search string:

Population (single terms): macrophyte*, seagrass, SAV, Charophyt*, Chara, Nitell*, Thalassia, Posidonia, Halophila, Zostera, Potamogeton, Myriophyllum, Ruppia, Ranunculus, Elodea, Ceratophyllum, Alisma, Hydrilla, Bryophyta, Utricularia, Nympha*, Nasturtium, Vallsneria, Cymodocea

Population (combined terms): aquatic, benthic, submerged, underwater *combined with* veget*, plant, *flora*, weed

Intervention: *boat*, *ferry*, ship*, watercraft*, berth*, mooring*, anchor*, wake*, propeller, pier, jetty, (wave NEAR/1 action), marina*

The terms within the two categories (‘population‘ and ‘intervention‘) were combined using the Boolean operator ‘OR’. The combined population terms and the categories were combined using the Boolean operator ‘AND’.The Boolean operator ‘NEAR’ was used to find the terms occurring together. An asterisk (*) represents any group of characters.

The intervention term ‘marina’ returned a large number of hits of publications on the seagrass *Zostera marina*. In order to include studies of boating marinas but not include all publications including this species, we constructed an additional search string replacing all intervention terms with marina* NOT “Zostera marina”.

The following search terms were tested but not included in the final search string, since they either did not return any relevant hits or returned a large number of irrelevant hits when added to the search string:

Population: Zannichellia, Najas, Stuckenia, Tolypella, Stratoides, Callitriche, Sagittaria, Sparganium, Cabomba, Nuphar, Heteranthera, Hippuris, Hottonia, Luronium, Batrachium, Groenlandia, Amphibolis, Enhalus, Halodule, Heterozostera, Phyllospadix, Syringodium, Thalassodendron

Intervention: jet-ski, navigation, jetties

Outcome: experiment*, comperative, BACI, (different AND pressure)

The outcome terms were not included in the string because they reduced the number of hits strongly. The number of hits based only on population and intervention was not larger than we could handle all of them.

The resulting full search strings were:

(macrophyte* OR seagrass OR SAV OR Charophyt* OR Chara OR Nitell* OR Thalassia OR Posidonia OR Halophila OR Zostera OR Potamogeton OR Myriophyllum OR Ruppia OR Ranunculus OR Elodea OR Ceratophyllum OR Alisma OR Hydrilla OR Bryophyta OR Utricularia OR Nympha* OR Nasturtium OR Vallisneria OR Cymodocea OR ((aquatic OR benthic OR submerged OR underwater) AND (vegetat* OR plant* OR *flora* OR weed))) AND (*boat* OR *ferry* OR ship* OR watercraft* OR berth* OR mooring* OR anchor* OR wake* OR propeller OR pier OR jetty OR (wave NEAR/1 action))

(macrophyte* OR seagrass OR SAV OR Charophyt* OR Chara OR Nitell* OR Thalassia OR Posidonia OR Halophila OR Zostera OR Potamogeton OR Myriophyllum OR Ruppia OR Ranunculus OR Elodea OR Ceratophyllum OR Alisma OR Hydrilla OR Bryophyta OR Utricularia OR Nympha* OR Nasturtium OR Vallisneria OR Cymodocea OR ((aquatic OR benthic OR submerged OR underwater) AND (vegetat* OR plant* OR *flora* OR weed))) AND marina* NOT ”Zostera marina”

Search outcome

The finalized search strings were used in the online citation databases; the ISI Web of Science and ASFA; Aquatic Science and Fisheries Abstracts, see results from the searches in Table S2 below. There is a bias in confining the search terms to English, but unfortunately, we did not have the resources to conduct searches in other languages. However, the search hits were not restricted to English. All publications in other languages returned from our searches have been translated and evaluated in the same way as the ones in English.

Table S2. Literature searches in the ISI Web of Science and Aquatic Science and Fisheries Abstracts (ASFA)

Search engine	Type of search	Search string	Comments	Date	No. of hits
ISI Web of Science	Search within the Topic field. No restriction in time span, source type, document type or language.	(macrophyte* OR seagrass OR SAV OR Charophyt* OR Chara OR Nitell* OR Thalassia OR Posidonia OR Halophila OR Zostera OR Potamogeton OR Myriophyllum OR Ruppia OR Ranunculus OR Elodea OR Ceratophyllum OR Alisma OR Hydrilla OR Bryophyta OR Utricularia OR Nympha* OR Nasturtium OR Vallisneria OR Cymodocea OR ((aquatic OR benthic OR submerged OR underwater) AND (vegetat* OR plant* OR *flora* OR weed))) AND (*boat* OR *ferry* OR ship* OR watercraft* OR berth* OR mooring* OR anchor* OR wake* OR propeller OR pier OR jetty OR (wave NEAR/1 action))	Final search string #1	30 Nov. 2015	995
ISI Web of Science	As above, but with time span from 2015	As above	Repeated search using final search string #1	20 Mar. 2019	316
ISI Web of Science	Search within the Topic field. No restriction in time span, source type, document type or language.	(macrophyte* OR seagrass OR SAV OR Charophyt* OR Chara OR Nitell* OR Thalassia OR Posidonia OR Halophila OR Zostera OR Potamogeton OR Myriophyllum OR Ruppia OR Ranunculus OR Elodea OR Ceratophyllum OR OR Alisma OR Hydrilla OR Bryophyta OR Utricularia OR Nympha* OR Nasturtium OR Vallisneria OR Cymodocea OR ((aquatic OR benthic OR submerged OR underwater) AND (vegetat* OR plant* OR *flora* OR weed))) AND marina* NOT "Zostera marina"	Final search string #2	30 Nov. 2015	446
ISI Web of Science	As above, but with time span from 2015	As above	Repeated search using final search string #2	20 Mar. 2019	62

Table S2. continued

Search engine	Type of search	Search string	Comments	Date	No. of hits
ASFA	Search within the Abstract field. No restriction in time span, source type or language. Books excluded from document types.	(macrophyte* OR seagrass OR SAV OR Charophyt* OR Chara OR Nitell* OR Thalassia OR Posidonia OR Halophila OR Zostera OR Potamogeton OR Myriophyllum OR Ruppia OR Ranunculus OR Elodea OR Ceratophyllum OR Alisma OR Hydrilla OR Bryophyta OR Utricularia OR Nympha* OR Nasturtium OR Vallisneria OR Cymodocea OR ((aquatic OR benthic OR submerged OR underwater) AND (vegetat* OR plant* OR *flora* OR weed))) AND (*boat* OR *ferry* OR ship* OR watercraft* OR berth* OR mooring* OR anchor* OR wake* OR propeller OR pier OR jetty OR (wave NEAR/1 action))	Final search string #1	30 Nov. 2015	958
ASFA	As above, but with time span from 1 Dec. 2015	As above	Repeated search using final search string #1	20 Mar. 2019	138
ASFA	Search within the Abstract field. No restriction in time span, source type or language. Books excluded from document types.	(macrophyte* OR seagrass OR SAV OR Charophyt* OR Chara OR Nitell* OR Thalassia OR Posidonia OR Halophila OR Zostera OR Potamogeton OR Myriophyllum OR Ruppia OR Ranunculus OR Elodea OR Ceratophyllum OR Alisma OR Hydrilla OR Bryophyta OR Utricularia OR Nympha* OR Nasturtium OR Vallisneria OR Cymodocea OR ((aquatic OR benthic OR submerged OR underwater) AND (vegetat* OR plant* OR *flora* OR weed))) AND marina* NOT "Zostera marina"	Final search string #2	30 Nov. 2015	297
ASFA	As above, but with time span from 1 Dec. 2015	As above	Repeated search using final search string #2	20 Mar. 2019	37

Database sub files included in the search

Both ISI Web of Science and ASFA give access to multiple databases that reference scientific literature. The databases covered by ASFA are fixed, while the coverage of Web of Science, in excess of their core collection, depends on the subscription treaty with Thomson Reuters, which may vary between universities and with time. A comprehensive list of all databases covered in the searches (2015-11-30 and 2019-03-20) are given below:

ISI Web of Science

- Science Citation Index Expanded (1945–2019)
- Social Sciences Citation Index (1956–2019)
- Arts & Humanities Citation Index (1975–2019)
- Conference Proceedings Citation Index- Science (1990–2019)
- Conference Proceedings Citation Index- Social Science & Humanities (1990–2019)
- Book Citation Index– Science (2005–2015)
- Book Citation Index– Social Sciences & Humanities (2005–2015)
- Emerging Sources Citation Index (2015–2019)
- MEDLINE (1950–2019)
- SciELO Citation Index (2002–2019)
- Korean Journal Index (1980–2019)
- Russian Science Citation Index (2005–2019)

ASFA

- Biological Sciences & Living Resources
- Ocean Technology, Policy & Non-Living Resources
- Aquatic Pollution & Environmental Quality
- Aquaculture Abstracts
- Marine Biotechnology Abstracts
- Oceanic Abstracts

Appendix S3. Excluded articles

Articles included based on title and abstract but not found in full text

Behie G, Cornillon P (1981) Mapping submerged aquatic vegetation in Chesapeake Bay. Uri, Narragansett, RI (USA).

Byfield A (1990) The Basingstoke Canal - Britain's richest waterway under threat. British Wildlife 2:13–21.

Fakiris E, Papatheodorou G, Geraga M, et al. (2009) Mapping of *P. oceanica* meadows using side-scan sonar and seafloor classification system (TextureAn): case studies of Laganas and Alikes bays (Zakynthos island, Greece). Hellenic Symposium on Oceanography and Fisheries Abstracts/Panellinio Symposio Okeanografias kai Alieias Perilipseis 1:128–133.

Lasagna R, Montefalcone M, Morri C, et al. (2008) Marine protected areas and *Posidonia oceanica* meadows: has the protection been effective till now? The case of the Portofino MPA (Ligurian sea). Atti dell'Associazione Italiana di Oceanologia e Limnologia 19:251–255.

Orth RJ, Frisch AA, Nowak JF, Moore K (1989) Distribution of submerged aquatic vegetation in the Chesapeake Bay and tributaries and Chincoteague Bay – 1987.

Powell C (2006) The (turtle) grass is always greener... Research project studies seagrass stressors, develops nondestructive test to determine photosynthetic health. Texas Shores 39:25–28.

Sun C-J, Gao Y-J, Cao Y, et al. (2010) Study on design and effect of estuary ecological floating bed pilot project at Dianshan lake. China Water & Wastewater 26:64–68.

Webb M, Locher U (1991) Intensive survey of rural and urban activities impacting water and coastal resources.

Articles excluded based on full text reading

Exclusion categories:

**Subj**	Excluded on subject population (no data on properties of vegetation)
**Act**	Excluded on activity (not a study of effects of boat traffic or infrastructure for mooring)
**Comp**	Excluded on comparator (no control treatment with no or low occurrence of the activity, or in some cases baseline studies before exploitation without any after data)
**Out**	Excluded on biological outcome (no data of cover, biomass or shoot density)
**Des**	Excluded on study design (e.g. studies based on simulation modelling, vegetation removal or transplantation; or un-replicated data)
**Rev**	No relevant primary data but potentially useful as a review

The reason for exclusion given below may in some cases be the first one identified by the reviewer rather than the most important one.

Ali MM, Murphy KJ, Langendorff J (1999) Interrelations of river ship traffic with aquatic plants in the River Nile, Upper Egypt. Hydrobiologia 415:93–100. **(Des)**

Allen MC, Read AJ (2000) Habitat selection of foraging bottlenose dolphins in relation to boat density near Clearwater, Florida. Marine Mammal Science 16:815–824. (**Des)**

Anderson LE, Manning RE, Monz CA, Goonan KA (2012) Indicators and standards of quality for paddling on Lake Champlain. Journal of Great Lakes Research 38:150–156. **(Subj)**

Andrade F, Ferreira MA (2011) A method for monitoring shallow seagrass meadows (*Zostera* spp.) using terrestrial oblique large-scale photography. Aquatic Botany 95:103–109. **(Act)**

Balaguer P, Diedrich A, Sarda R, et al. (2011) Spatial analysis of recreational boating as a first key step for marine spatial planning in Mallorca (Balearic Islands, Spain). Ocean & Coastal Management 54:241–249. **(Act)**

Balduzzi A, Bianchi CN, Cattaneo Vietti R, et al. (1994) First data on the benthic communities around the Gallinaria Island (Ligurian sea, NW Mediterranean Sea). Atti dell'Associazione Italiana di Oceanologia e Limnologia 10:603–617. **(Subj)**

Bishop M (2004) A posteriori evaluation of strategies of management: The effectiveness of no-wash zones in minimizing the impacts of boat-wash on macrobenthic infauna. Environmental Management 34:140–149. **(Subj)**

Bishop M (2008) Displacement of epifauna from seagrass blades by boat wake. Journal of Experimental Marine Biology and Ecology 354:111–118. **(Subj)**

Bishop MJ (2005) Compensatory effects of boat wake and dredge spoil disposal on assemblages of macroinvertebrates. Estuaries 28:510–518. **(Subj)**

Boese BL, Kaldy JE, Clinton PJ, et al. (2009) Recolonization of intertidal *Zostera marina* L. (eelgrass) following Experimental shoot removal. Journal of Experimental Marine Biology and Ecology 374:69–77. **(Des)**

Bonham AJ (1983) The management of wave-spending vegetation as bank protection against boat wash. Landscape Planning 10:15–30. **(Subj)**

Borman S, Bachmann R, Jones J, et al. (eds) (1995) The benefits of trend monitoring for macrophyte management: A case study. Lake and Reservoir Management 11:119. **(Out)**

Boudouresque C-F (2004) Marine biodiversity in the Mediterranean: status of species, populations and communities. Travaux scientifiques du Parc national de Port-Cros 20:97–146. **(Rev)**

Boudouresque CF, Bernard G, Pergent G, et al. (2009) Regression of Mediterranean seagrasses caused by natural processes and anthropogenic disturbances and stress: a critical Review. Botanica Marina 52:395–418. **(Comp)**

Brock R, Culhane B (2004) The no-take research natural area of dry Tortugas National Park (Florida): Wishful thinking or responsible planning? In: Shipley, JB (ed) Aquatic protected areas as fisheries management tools. Amer Fisheries Soc; Natl Sea Grant Coll, pp 67–74. **(Comp)**

Browne NK, Yaakub SM, Tay JKL, Todd PA (2017) Recreating the shading effects of ship wake induced turbidity to test acclimation responses in the seagrass Thalassia hemprichii. Estuarine Coastal and Shelf Science 199:87–95. **(Act)**

Bryan M, Scarnecchia DL (1992) Species richness, Composition, and abundance of fish larvae and juveniles inhabiting natural and developed shorelines of a glacial Iowa lake. Environmental Biology of Fishes The Hague 35:329–341. **(Subj)**

Buchan L, Padilla DK (2000) Predicting the likelihood of Eurasian watermilfoil presence in lakes, a macrophyte monitoring tool. Ecological Applications 10:1442–1455. **(Des)**

Burfeind DD, Stunz GW (2007) The effects of boat propeller scarring on nekton growth in subtropical seagrass meadows. Transactions of the American Fisheries Society 136:1546–1551. **(Subj)**

Byrnes T, Warnken J (2003) Establishing best-practice environmental management: Lessons from the Australian tour-boat industry. In: Buckley, R and Pickering, C and Weaver, DB (ed) Nature-based tourism, environment and land management. Ctr Ecotourism Res; Australian Acad Sci; Griffith Univ, pp 111–121. **(Subj)**

Carter GA, Lucas KL, Biber PD, et al. (2011) Historical changes in seagrass coverage on the Mississippi barrier islands, northern Gulf of Mexico, determined from vertical aerial imagery (1940-2007). Geocarto International 26:663–673. **(Comp)**

Casalta B (2010) Impact of the anchoring of big ships on the *Posidonia oceanica* meadow in Var (Provence, France). Travaux scientifiques du Parc national de Port-Cros 24:205–207. **(Act)**

Ceccherelli G, Campo D (2002) Experimental evaluation of anchor damage on *Posidonia oceanica* L. (Delile). Biologia marina mediterranea 9:672–673. **(Act)**

Ceccherelli G, Campo D, Milazzo M (2007) Short-term response of the slow growing seagrass *Posidonia oceanica* to simulated anchor impact. Marine Environmental Research 63:341–349. **(Des)**

Ceccherelli G, Pinna S, Cusseddu V, Bulleri F (2014) The role of disturbance in promoting the spread of the invasive seaweed *Caulerpa racemosa* in seagrass meadows. Biological Invasions 16:2737–2745. **(Des)**

Chaouti A, Bayed A (2005) The effects of anthropogenic perturbation on the hydrology of Lake Smir (NW, Morocco) (Nord-Ouest, Maroc). Revue des sciences de l'eau/journal of water science 18:181–197. **(Act)**

Chen S-Y (2017) Flow-resistance adaptations of rigid-stemmed aquatic macrophytes in simulated water channels. Landscape and Ecological Engineering 13:157–167. **(Act)**

Clark PA (1995) Evaluation and management of propeller damage to seagrass beds in Tampa Bay, Florida. Florida Scientist 58:193–196. **(Subj)**

Collier KJ, Clapcott JE, David BO, et al. (2013) Macroinvertebrate-Pressure Relationships in Boatable New Zealand Rivers: Influence of Underlying Environment and Sampling Substrate. River Research and Applications 29:645–659. **(Act)**

Collins KJ, Suonp AM, Mallinson JJ (2010) The impacts of anchoring and mooring in seagrass, Studland Bay, Dorset, UK. Underwater Technology 29:117–123. **(Subj)**

Collins N, St. Onge P, Dodington V, et al. (eds) (1995) The importance to small fish of littoral fringe habitat (Z < 0.2 m) in unproductive lakes, and the impacts of shoreline development. Lake and Reservoir Management 11:129. **(Subj)**

Cordell JR, Munsch SH, Shelton ME, Toft JD (2017) Effects of piers on assemblage composition, abundance, and taxa richness of small epibenthic invertebrates. Hydrobiologia 802:211–220. **(Subj)**

Crawford RE, Stolpe NE, Moore M (1998) Environmental impacts of boating. Proceedings of a workshop held at Woods Hole Oceanographic Institution, Woods Hole, Massachusetts on December 7-9, 1994. **(Subj)**

Creed J, Amado G (1999) Disturbance and recovery of the macroflora of a seagrass (*Halodule wrightii* Ascherson) meadow in the Abrolhos Marine National Park, Brazil: an Experimental evaluation of anchor damage. Journal of Experimental Marine Biology and Ecology 235:285–306. **(Des)**

Creed JC (2000) The biodiversity of Brazil's seagrasses and seagrass habitats: a first analysis. Biologia marina mediterranea 7:207–210. **(Act)**

Crigger D, Graves G, Fike D (2005) Lake Worth Lagoon conceptual ecological model. Wetlands 25:943–954. **(Subj)**

Cruz-Palacios V, Van Tussenbroek BI (2005) Simulation of hurricane-like disturbances on a Caribbean seagrass bed. Journal of Experimental Marine Biology and Ecology 324:44–60. **(Act)**

Cunha AH, Erzini K, Serrao EA, et al. (2014) Biomares, a LIFE project to restore and manage the biodiversity of Prof. Luiz Saldanha Marine Park. Journal of Coastal Conservation 18:643–655. **(Subj)**

Cuttriss AK, Prince JB, Castley JG (2013) Seagrass communities in southern Moreton Bay, Australia: Coverage and fragmentation trends between 1987 and 2005. Aquatic Botany 108:41–47. **(Comp)**

D'Antuono K (2000) The National Park Service's proposed ban: A new approach to personal watercraft use in the National Parks. Boston College Environmental Affairs Law Review 27:243–278. **(Subj)**

Dawes CJ, Andorfer J, Rose C, et al. (1997) Regrowth of the seagrass *Thalassia testudinum* into propeller scars. Aquatic Botany 59:139–155. **(Des)**

De Biasi A, Torricelli L, Aliani S (1997) The *Posidonia oceanica* meadow of Portovenere (eastern Ligurian Sea). Biologia marina mediterranea 4:500–501. **(Comp)**

De Falco G, Ferrari S, Cancemi G, Baroli M (2000) Relationship between sediment distribution and *Posidonia oceanica* seagrass. Geo-Marine Letters 20:50–57. **(Act)**

De Muro S, Pusceddu N, Buosi C, Ibba A (2017) Morphodynamics of a Mediterranean microtidal wave-dominated beach: forms, processes and insights for coastal management. Journal of Maps 13:26–36. **(Subj)**

de Visser A, Boeters R (1997) Designing ecologically sound banks along navigation canals. In: Wang, SSY and Carstens, T (ed) Environmental and coastal hydraulics: protecting the aquatic habitat, proceedings of theme b, vols 1 & 2. Int Assoc Hydraul Res, pp 498–503. **(Subj)**

Deis D (2000) Monitoring the effects of construction and operation of a marina on the seagrass *Halophila decipiens* in Fort Lauderdale, Florida. In: Bortone, SA (ed) Seagrasses: monitoring, ecology, physiology, and management. pp 147–155. **(Comp)**

Desmonts D, Fritz H, Cornulier T, Maheo R (2009) Rise in human activities on the mudflats and Brent Geese (*Branta bernicla*) wintering distribution in relation to *Zostera* spp. beds: a 30-year study. Journal of Ornithology 150:733–742. **(Act)**

Diedrich A, Balaguer Huguet P, Tintore Subirana J (2011) Methodology for applying the Limits of Acceptable Change process to the management of recreational boating in the Balearic Islands, Spain (Western Mediterranean). Ocean & Coastal Management 54:341–351. **(Subj)**

Diedrich A, Terrados J, Arroyo NL, Balaguer P (2013) Modeling the influence of attitudes and beliefs on recreational boaters' use of buoys in the Balearic Islands. Ocean & Coastal Management 78:112–120. **(Subj)**

Diener RA (1975) Cooperative Gulf of Mexico estuarine inventory and study-Texas: area description. Circular / National Oceanic and Atmospheric Administration 393:1–129. **(Act)**

Duarte CM (2002) The future of seagrass meadows. Environmental Conservation 29:192–206. **(Rev)**

Duigan C, Reid S, Monteith DT, et al. (1999) The past, present and future of Llangorse Lake - a shallow nutrient-rich lake in the Brecon Beacons National Park, Wales, UK. Aquatic Conservation: Marine and Freshwater Ecosystems 9:329–341. **(Act)**

Engeman RM, Duquesnel JA, Cowan EM, et al. (2008) Assessing boat damage to seagrass bed habitat in a Florida Park from a bioeconomics perspective. Journal of Coastal Research 24:527–532. **(Out)**

Fonseca M, Whitfield P, Kenworthy W, et al. (2004) Use of two spatially explicit models to determine the effect of injury geometry on natural resource recovery. Aquatic Conservation-Marine and Freshwater Ecosystems 14:281–298. **(Des)**

Francour P, Ganteaume A, Poulain M (1999) Effects of boat anchoring in *Posidonia oceanica* seagrass beds in the Port-Cros National Park (north-western Mediterranean Sea). Aquatic Conservation: Marine and Freshwater Ecosystems 9:391–400. **(Act)**

Fresh KL, Wyllie-Echeverria T, Wyllie-Echeverria S, Williams BW (2006) Using light-permeable grating to mitigate impacts of residential floats on eelgrass *Zostera marina* L. in Puget Sound, Washington. Ecological Engineering 28:354–362. **(Des)**

Gambi MC, Barbieri F (2013) Regression of *Halophila stipulacea* (Forssk.) Aschers. (Hydrocharitaceae) in the harbour of Palinuro (Salerno, Italy). Biologia marina mediterranea 20:134–135. **(Act)**

Ganteaume A, Bonhomme P, Bernard G, et al. (2005a) Pleasure boats anchoring impact on the *Posidonia oceanica* meadow in the National park of Port-Cros (North-Western Mediterranean). Travaux scientifiques du Parc national de Port-Cros 21:147–162. **(Act)**

Ganteaume A, Bonhomme P, Emery E, et al. (2005b) Cruising boats mooring impact on the *Posidonia oceanica* meadow, on the island of Porquerolles (Provence, France, Mediterranean). Travaux scientifiques du Parc national de Port-Cros 21:163–173. **(Act)**

Gerard P, De Bast B (2000) Restriction of the circulation of small pleasure boats on the rivers of Wallonia, Belgium. Fisheries Management and Ecology 7:139–143. **(Act)**

Giani L, Cossu A (2003) Stress of anthropic effects and ecological conditions of the *Posidonia oceanica* fields in the Maddalena National Park (NE Sardinia). Biologia Marina Mediterranea 10:711–713. **(Comp)**

Gibbs P (1997) Botany Bay seagrass habitat restoration. Fisheries NSW Sydney 1:17–18. **(Subj)**

Glemarec M, LeFaou Y, Cuq F (1997) Long-term changes of seagrass beds in the Glenan Archipelago (South Brittany). Oceanologica Acta 20:217–227. **(Act)**

Goulder R (2003) Aquatic plants in the Pocklington and Driffield Canals with regard to the potential extension of navigation. Naturalist 128:129–146. **(Out)**

Goulder R (2008) Conservation of aquatic plants in artificial watercourses: are main drains a substitute for vulnerable navigation canals? Aquatic Conservation: Marine and Freshwater Ecosystems 18:163–174. **(Act)**

Green K, Tukman M, Finkbelner M (2011) Comparison of DMC, UltraCam, and ADS40 Imagery for benthic habitat and propeller scar mapping. Photogrammetric Engineering and Remote Sensing 77:589–599. **(Comp)**

Guala I, Brundu G, Vallainc D, Mossone P (2013) Assessment of conservation status of *Posidonia oceanica* and anchoring pressure as a tool for a proper management of recreational boating. Biologia Marina Mediterranea 20:164–165. **(Comp)**

Hallac DE, Sadle J, Pearlstine L, et al. (2012) Boating impacts to seagrass in Florida Bay, Everglades National Park, Florida, USA: links with physical and visitor-use factors and implications for management. Marine & Freshwater Research 63:1117–1128. **(Comp)**

Hammerstrom KK, Kenworthy WJ, Whitfield PE, Merello MF (2007) Response and recovery dynamics of seagrasses *Thalassia testudinum* and *Syringodium filiforme* and macroalgae in experimental motor vessel disturbances. Marine Ecology Progress Series 345:83–92. **(Des)**

Han Q, Bouma TJ, Brun FG, et al. (2012) Resilience of *Zostera noltii* to burial or erosion disturbances. Marine Ecology Progress Series 449:133–143. **(Des)**

Hannan J, Simpson A (1999) Scarred seagrass. Fisheries NSW Sydney 2:32. **(Subj)**

Harding W (1994) Water-quality trends and the influence of salinity in a highly regulated estuary near Cape-Town, South-Africa. South African Journal of Science 90:240–246. **(Subj)**

Hastings K, Hesp P, Kendrick GA (1995) Seagrass loss associated with boat moorings at Rottnest Island, Western Australia. Ocean & Coastal Management 26:225–246. **(Comp)**

Henricson C, Sandberg-Kilpi E, Munsterhjelm R (2006) Experimental studies on the impact of turbulence, turbidity and sedimentation on *Chara tomentosa* L. Cryptogamie: algologie 27:419–434. **(Out)**

Hertler H, Spotila J, Kreeger DA (2004) Effects of houseboats on organisms of the La Parguera reserve, Puerto Rico. Environmental Monitoring and Assessment 98:391–407. **(Comp)**

Holon F, Mouquet N, Boissery P, et al. (2015) Fine-scale cartography of human impacts along French Mediterranean coasts: a relevant map for the management of marine ecosystems. Plos One 10:e0135473. **(Des)**

Hutchinson N, Dodington V, Stirling M, et al. (eds) (1995) Shoreline alterations on the Muskoka Lakes, Ontario -- mapping techniques, methods development, preliminary results and interpretive framework. Lake and Reservoir Management 11:151. **(Subj)**

Iannuzzi T, Weinstein M, Sellner K, Barrett J (1996) Habitat disturbance and marina development: An assessment of ecological effects.1. Changes in primary production due to dredging and marina construction. Estuaries 19:257–271. **(Des)**

Johansson J, Greening H (2000) Seagrass restoration in Tampa Bay: A resource-based approach to estuarine management. In: Bortone, SA (ed) Seagrasses: monitoring, ecology, physiology, and management. pp 279–293. **(Comp)**

Kennison G, Dunsford DS, Schutten J (1998) Stable or changing lakes? A classification of aquatic macrophyte assemblages from a eutrophic shallow lake system in the United Kingdom. Aquatic Conservation: Marine and Freshwater Ecosystems 8:669–684. **(Act)**

Kenworthy Wj, Hammerstrom KK, Fonseca MS (2006) Scientific evaluation of a sediment fill technique for the restoration of motor vessel injuries in seagrass beds of the Florida Keys National Marine Sanctuary. National Oceanic and Atmospheric Administration. **(Des)**

Kininmonth S, Lemm S, Malone C, Hatley T (2014) Spatial vulnerability assessment of anchor damage within the Great Barrier Reef World Heritage Area, Australia. Ocean & Coastal Management 100:20–31. **(Des)**

Kirsch K, Barry K, Fonseca M, et al. (2005) The mini-312 program - An expedited damage assessment and restoration process for seagrasses in the Florida Keys National Marine Sanctuary. Journal of Coastal Research 109–119. **(Des)**

La Nafie YA, de los Santos CB, Brun FG, et al. (2012) Waves and high nutrient loads jointly decrease survival and separately affect morphological and biomechanical properties in the seagrass *Zostera noltii*. Limnology and Oceanography 57:1664–1672. **(Act)**

Larkin PD, Heideman KL, Burfeind DD, Stunz GW (2010) The effect of boat propeller scarring intensity on genetic variation in a subtropical seagrass species. Botanica Marina 53:99–102. **(Out)**

Lathrop RG, Haag SM, Merchant D, et al. (2014) Comparison of remotely-sensed surveys vs. in situ plot-based assessments of sea grass condition in Barnegat Bay-Little Egg Harbor, New Jersey USA. Journal of Coastal Conservation 18:299–308. **(Act)**

Leriche A, Pasqualini V, Boudouresque C-F, et al. (2006) Spatial, temporal and structural variations of a *Posidonia oceanica* seagrass meadow facing human activities. Aquatic Botany 84:287–293. **(Comp)**

Lipkin Y (1972) Vegitation of the Bitter Lakes in the Suez Canal water system. Israel Journal of Zoology 21:447–457. **(Act)**

Liston CR, McNabb CD, Duffy W, et al. (1983) Environmental baseline studies of St. Marys River near Neebish Island, Michigan, prior to proposed extension of navigation season, 1981. Great Lakes-St. Lawrence Seaway, Navigation Season Extension Program. **(Comp)**

Lloret J, Riera V (2008) Evolution of a Mediterranean coastal zone: human impacts on the marine environment of Cape Creus. Environmental Management 42:977–988. **(Subj)**

Lloret J, Zaragoza N, Caballero D, Riera V (2008) Impacts of recreational boating on the marine environment of Cap de Creus (Mediterranean sea). Ocean & Coastal Management 51:749–754. **(Act)**

Lorenz S, Pusch MT, Blaschke U (2015) Minimum shoreline restoration requirements to improve the ecological status of a north-eastern German glacial lowland lake in an urban landscape. Fundamental and Applied Limnology 186:323–332. **(Subj)**

Macreadie PI, York PH, Sherman CDH, et al. (2014a) No detectable impact of small-scale disturbances on `blue carbon' within seagrass beds. Marine Biology 161:2939–2944. **(Subj)**

Macreadie PI, York PH, Sherman CDH (2014b) Resilience of *Zostera muelleri* seagrass to small-scale disturbances: the relative importance of asexual versus sexual recovery. Ecology and Evolution 4:450–461. **(Des)**

Malicky G (1981) Quantitative data on the macrophytes of the Mayrbucht 1980. Jahresbericht Biologische Station Lunz der Osterreichischen Akademie der Wissenschaften Lunz 4(1980):177–181. **(Act)**

Maragos J, Cook CJ (1995) The 1991-1992 rapid ecological assessment of Palau’s coral reefs. Coral Reefs 14:237–252. **(Act)**

Martin SR, Onuf CP, Dunton KH (2008) Assessment of propeller and off-road vehicle scarring in seagrass beds and wind-tidal flats of the southwestern Gulf of Mexico. Botanica Marina 51:79–91. **(Comp)**

Menesatti P, Urbani G, Dolce T (2007) Spectrophotometric system to develop a non-invasive method for monitoring of *Posidonia oceanica* Meadows - art. no. 676119. In: Chen, YR and Meyer, GE (ed) Optics for natural resources, agriculture, and foods II. SPIE, p 76119. **(Act)**

Milazzo M, Badalamenti F, Ceccherelli G, Chemello R (2004) Boat anchoring on *Posidonia oceanica* beds in a marine protected area (Italy, western Mediterranean): effect of anchor types in different anchoring stages. Journal of Experimental Marine Biology and Ecology 299:51–62. **(Comp)**

Milazzo M, Badalamenti F, Riggio S, Chemello R (2002) Effects of boat anchoring in the *Posidonia oceanica* meadow of the Ustica Island Marine Reserve: preliminary data. Biologia Marina Mediterranea 9:686–689. **(Comp)**

Moss B (1977) Conservation problems in the Norfolk Broads and rivers of East Anglia, England—phytoplankton, boats and the causes of turbidity. Biol Conserv, 12(2), 95-114, (1977). **(Subj)**

Nelson TA, Gillanders SN, Harper J, Morris M (2011) Nearshore aquatic habitat monitoring: a seabed imaging and mapping approach. Journal of Coastal Research 27:348–355. **(Out)**

Nielsen A (1990a) Coupon Bight Aquatic Preserve management plan. **(Subj)**

Nielsen A (1990b) Lignumvitae Key Aquatic Preserve management plan. **(Subj)**

Oguslu E, Erkanli S, Hill VJ, et al. (2014) Detection of seagrass scars using sparse coding and morphological filter. In: Bostater, CR and Mertikas, SP and Neyt, X (ed) Remote sensing of the ocean, sea ice, coastal waters, and large water regions 2014. SPIE. **(Act)**

Okudan ES, Demir V, Kalkan E, Karhan Su (2011) Anchoring damage on seagrass meadows (*Posidonia oceanica* (L.) Delile) in Fethiye-Gocek specially protected area (eastern Mediterranean sea, Turkey). Journal of Coastal Research 417–420. **(Act)**

Pasqualini V, Pergent-Martini C, Pergent G (1999) Environmental impact identification along the Corsican coast (Mediterranean sea) using image processing. Aquatic Botany 65:311–320. **(Des)**

Patriquin DG (1975) “Migration” of blowouts in seagrass beds at Barbados and Carriacou, West Indies, and its ecological and geological implications. Aquatic Botany 1:163–189. **(Act)**

Pinder L (1997) Research on the Great Ouse: Overview and implications for management. Regulated Rivers-Research & Management 13:309–315. **(Rev)**

Pitanga ME, Montes MJF, Magalhaes KM, Reis TNV (2012) Quantification and classification of the main environmental impacts on a *Halodule wrightii* seagrass meadow on a tropical island in northeastern Brazil. Anais Da Academia Brasileira De Ciencias 84:35–42. **(Subj)**

Pitkaenen H, Peuraniemi M, Westerbom M, et al. (2013) Long-term changes in distribution and frequency of aquatic vascular plants and charophytes in an estuary in the Baltic Sea. Annales Botanici Fennici 50:1–54. **(Act)**

Ragazzola F, Cossu A, Mulargia M, et al. (2005) Extension and conservation of *Posidonia oceanica* (L.) Delile meadow in front of the Spiaggia Rosa (Budelli Island). Biologia marina mediterranea 12:151–154. **(Act)**

Rasheed M (1999) Recovery of experimentally created gaps within a *tropical Zostera capricorni* (Aschers.) seagrass meadow, Queensland Australia. Journal of Experimental Marine Biology and Ecology 235:183–200. **(Des)**

Robert P (1983) Degradation of the Posidonia sea-grass in the organized anchorage area of Port-Cros Bay. Travaux scientifiques du Parc national de Port-Cros Hyeres 9:195–197. **(Cont)**

Roca G, Romero J, Columbu S, et al. (2014) Detecting the impacts of harbour construction on a seagrass habitat and its subsequent recovery. Ecological Indicators 45:9–17. **(Act)**

Rogers CS, Beets J (2001) Degradation of marine ecosystems and decline of fishery resources in marine protected areas in the US Virgin Islands. Environmental Conservation 28:312–322. **(Comp)**

Sbrescia L, De Vita A, Di Stefano F, et al. (2012) Thematic cartography to choose mooring and anchoring areas in Santa Maria di Castellabate MPA. Biologia Marina Mediterranea 19:114–115. **(Act)**

Serrano O, Ruhon R, Lavery PS, Kendrick GA, Hickey S, Masque P, Arias-Ortiz A, Steven A, Duarte CM (2016) Impact of mooring activities on carbon stocks in seagrass meadows. Scientific Reports 6:23193. **(Out)**

Sidman C, Antonini G, Sauers S, et al. (2000) Evaluating recreational boating patterns at selected sites in southwest Florida for regional anchorage management. **(Subj)**

Simenstad C, Thom RM, Kuzis K, et al. (1988) Nearshore community studies of Neah Bay, Washington. Final contract report to U.S. Army Corps of Engineers, Seattle District Environmental Resources Section, Seattle, Washington. **(Act)**

Simenstad C, Thom RM, Olson AM eds. (1997) Mitigating potential impacts of ferry terminal siting and design on eelgrass habitat. Research Report, Washington State Transportation Centre. **(Comp)**

Simons J, Bakker C, Schropp M, et al. (2001) Man-made secondary channels along the River Rhine (the Netherlands); results of post-project monitoring. Regulated Rivers-Research & Management 17:473–491. **(Subj)**

Smith KA, North EW, Shi F, et al. (2009) Modeling the effects of oyster reefs and breakwaters on seagrass growth. Estuaries and Coasts 32:748–757. **(Des)**

Stuckey RL (1971) Changes in vascular aquatic flowering plants during 70 years in Put-in-Bay Harbour, Lake Erie, Ohio. Ohio J Sci 71:321–342. **(Out)**

Sukopp H (1971) Effects of man, especially recreational activities, on littoral macrophytes. Hidrobiologia, Bucur 12:331–340. **(Subj)**

Sutherland TF, Elner RW, O'Neill JD (2013) Roberts Bank: Ecological crucible of the Fraser River estuary. Progress in Oceanography 115:171–180. **(Act)**

Sutherland TF, Morrison S, Peterson S, et al. (2006) Data report on the distribution and density of two eelgrass species, *Zostera marina* and *Zostera japonica,* on Roberts Bank, British Columbia. **(Act)**

Thom R, Williams G, Borde A, et al. (2005) Adaptively addressing uncertainty in estuarine and near coastal restoration projects. Journal of Coastal Research 94–108. **(Des)**

Toft J, Simenstad C, Young C, Stamatiou L (2003) Inventory and mapping of city of Seattle shorelines along Lake Washington, the ship canal, and Shilshole bay. Technical report School of Aquatic and Fishery Science, Fisheries Research Institute, Washington University. **(Act)**

Tolonen KT, Haemaelaeinen H, Holopainen IJ, Karjalainen J (2001) Influences of habitat type and environmental variables on littoral macroinvertebrate communities in a large lake system. Archiv fuer Hydrobiologie 152:39–67. **(Act)**

Torn K, Martin G (2009) Effect of mechanical disturbances on the charophyte community in the northern Baltic Sea. Phycologia 48:131. **(Des)**

Tuya F, Sanchez-Jerez P, Dempster T, et al. (2006) Changes in demersal wild fish aggregations beneath a sea-cage fish farm after the cessation of farming. Journal of Fish Biology 69:682–697. **(Act)**

Uhrin AV, Holmquist JG (2003) Effects of propeller scarring on macrofaunal use of the seagrass *Thalassia testudinum*. Marine Ecology Progress Series 250:61–70. **(Subj)**

Vazquez-Luis M, Borg JA, Morell C, et al. (2015) Influence of boat anchoring on *Pinna nobilis*: a field experiment using mimic units. Marine and Freshwater Research 66:786–794. **(Act)**

Wade PM (1999) The impact of human activity on the aquatic macroflora of Llangorse Lake, South Wales. Aquatic Conservation: Marine and Freshwater Ecosystems 9:441–459. **(Act)**

Walker D, Lukatelich RJ, Bastyan G, McComb AJ (1989) Effect of boat moorings on seagrass beds near Perth, Western Australia. Aquatic Botany 36:69–77. **(Out)**

Wang P, Beck TM (2017) Determining Dredge-Induced Turbidity and Sediment Plume Settling within an Intracoastal Waterway System. Journal of Coastal Research 33:243–253. **(Subj)**

Weber A, Lautenbach S, Wolter C (2012) Improvement of aquatic vegetation in urban waterways using protected artificial shallows. Ecological Engineering 42:160–167. **(Des)**

Weber A, Garcia X-F, Wolter C (2017) Habitat rehabilitation in urban waterways: the ecological potential of bank protection structures for benthic invertebrates. Urban Ecosystems 20:759–773. **(Subj)**

Weber A, Wolter C (2017) Habitat rehabilitation for juvenile fish in urban waterways: A case study from Berlin, Germany Journal of Applied Ichthyology/Zeitschrift fur angewandte Ichthyologie 33:136–143. **(Subj)**

Wetherell V, Nielsen A (1991) Biscayne Bay Card Sound: Aquatic preserve management plan. **(Subj)**

Whitfield AK, Becker A (2014) Impacts of recreational motorboats on fishes: A review. Marine Pollution Bulletin 83:24–31. **(Rev)**

Widmer WM (2006) Using the precautionary principle to measure recovery of coastal habitats: The case of a seagrass bed. Journal of Coastal Research 962–965. **(Des)**

Williams SL (1988) *Thalassia testudinum* productivity and grazing by green turtles in a highly disturbed seagrass bed. Marine Biology Berlin, Heidelberg 98:447–455. **(Act)**

Yousef M, Nordheim von H, Schubert H (2002) Distribution, threat status and conservation of charophytes in the inner coastal waters of Mecklenburg-Western Pomerania (southern Baltic Sea). Natur und Landschaft 77:304–309. **(Subj)**

Zieman JC (1976) The ecological effects of physical damage from motor boats on turtle grass beds in southern Florida. Aquatic Botany 2:127–139. **(Subj)**

Articles included based on full text reading but excluded based on critical appraisal

Exclusion categories:

**Meth**	Excluded on methodological detail (methodology not described)
**Cont**	Inappropriate control (comparison between different depth ranges, different seasons, or comparison difficult to interpret for the purposes of this review)
**Expi**	Exposure data difficult to interpret (conclusion on exposure intensity based on the level of vegetation damage or dubious link between boating activity and vegetation properties)
**Red**	Excluded on redundancy (primary data also published elsewhere)

The reason for exclusion given below may in some cases be the first one identified by the reviewer rather than the most important one.

Bell SS, Hall M, Soffian S, Madley K (2002) Assessing the impact of boat propeller scars on fish and shrimp utilizing seagrass beds. Ecological Applications 12:206–217. **(Cont)**

Burfeind DD, Stunz GW (2006) The effects of boat propeller scarring intensity on nekton abundance in subtropical seagrass meadows. Marine Biology 148:953–962. **(Expi)**

Fonseca ACE, Nielsen VM, Cortes J (2007) Seagrass monitoring at Perezoso, Cahuita, Costa Rica (CARICOMP site). Revista de Biologia Tropical 55:55–66. **(Expi)**

Hendriks IE, Tenan S, Tavecchia G, et al. (2013) Boat anchoring impacts coastal populations of the pen shell, the largest bivalve in the Mediterranean. Biological Conservation 160:105–113. **(Meth)**

Montefalcone M, Lasagna R, Bianchi CN, et al. (2006) Anchoring damage on *Posidonia oceanica* meadow cover: a case study in Prelo Cove (Ligurian Sea, NW Mediterranean). Chemistry and ecology 22:S207–S217. **(Cont)**

Mumma MT, Cichra CE, Sowards JT (1996) Effects of recreation on the submersed aquatic plant community of Rainbow River, Florida. Journal of Aquatic Plant Management 34:53–56. **(Cont)**

Willby NJ, Eaton JW (1996) Backwater habitats and their role in nature conservation on navigable waterways. Hydrobiologia 340:333–338. **(Red)**

Appendix S4. Handling of dependent data

When single articles reported comparison of means from more than one site or survey (i.e. surveys conducted in the field at different occasions and/or in different sites), we included these as separate effect sizes in the analyses. Other types of dependent data (such as, multiple readings from different depths or habitats within the same site, or data reported from multiple time points, multiple measures of vegetation abundance or multiple treatments) were merged into composite scores before analysis (Borenstein et al. 2009), as described in the following paragraphs.

When a study reported data from different depths or habitats within the same site, they were treated as independent subgroups and the single effect sizes and their variances were combined into composites using a fixed-effect model.

When more than one measure of vegetation abundance was reported from the same study (e.g., shoot density and biomass), the effect sizes from the different measures were combined into a single composite through averaging. The variance (V_{mean}) of the composite effect size was calculated using the following equation;

$$V_{mean} = \left(\sum_{i=1}^{m} V_i + \ 2 \sum_{i,j} \left(r_{ij} \sqrt{V_i} \sqrt{V_j} \right) \right) \Big/ m^2$$

where V_i is the variance for the effect size of each outcome, m is the number of outcomes within the study and r is the correlation between two outcomes. The correlations were estimated from data reported within the same study or from data reported in other studies of the same vegetation species within the same geographic area.

When a study reported data collected repeatedly within the same year but over different seasons a composite effect size was calculated as described above, to reduce seasonal effects associated with the time of sampling. Whenever data was reported from more than one treatment sharing the same control or from one impacted site compared to several controls, a composite effect size was calculated as described above; with the difference that r was set to 0.5. The reason for this is that half of the information is redundant when merging two effect sizes calculated from the same control, since the data of the control is entered twice.

Reference

Borenstein, M., L. V. Hedges, J. Higgins, and H. R. Rothstein. 2009. *Introduction to Meta-Analysis*. West Sussex, UK: Wiley.

Appendix S5. Assessment of publication bias

Publication bias, i.e., the lack of small studies with small non-significant effects, can generally be discovered through visual inspection of funnel plots (Borenstein et al. 2009; Koricheva et al. 2013). The inverted relationship between the standard error and study size distributes the studies according to size along the y-axis. In the absence of publication bias, a symmetric distribution of effects is expected along the x-axis, with a decreasing spread towards the top of the figure, as the larger studies yields more accurate estimates. However, the small number of studies makes the funnel plots hard to interpret (Fig. S1a-c). Thus, publication bias could neither be confirmed nor ruled out by the aid of funnel plots.

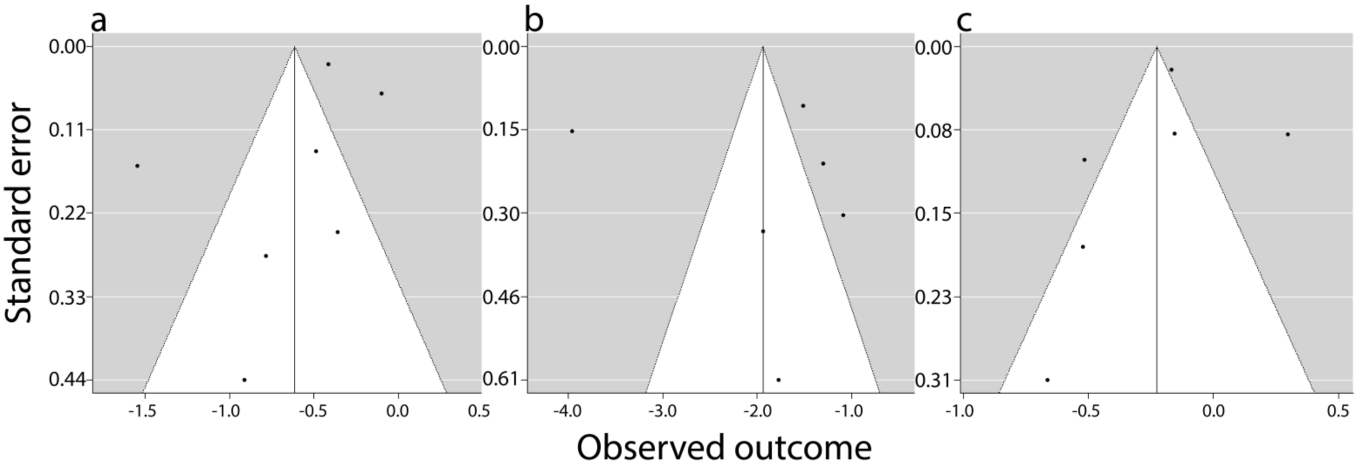

Figure S1. Funnel plots for the data sets of a) boat traffic, b) docks and c) mooring areas.

However, Rosenthal´s fail-safe number calculation could be used to assess whether the entire observed effect could be an artefact, through calculating how many non-published studies with a mean effect of zero it takes to nullify the summary effect (Rosenthal 1979; Begg and Mazumdar 1994). Rosenthal´s fail-safe number calculation showed that it would take 555 and 1396 "missing" studies with a mean effect of zero to nullify the summary effect for the data sets on boat traffic and docks respectively, indicating that the summary effect is unlikely to be an artefact of bias. For mooring areas the fail-safe number was 88, still indicating that the summary effect is unlikely to be an artefact of bias, but with more uncertainty than for the other two exposure categories.

Further, cumulative meta-analysis with studies sorted by sample size was used to explore whether small studies with large effect size influenced the summary effects (Borenstein et al. 2009). For the data sets on boat traffic and docks, the analyses showed that the summary effects were not inflated by small studies (Table S3). Thus, we conclude that a possible publication bias is unlikely to affect the summary effects. However, for mooring areas the analysis showed that the summary effects was inflated by small studies and hence there is a risk that the summery effect is influenced by a publication bias.

Table S3. Cumulative meta-analysis exploring how smaller studies affects the effect size estimate for a) boat traffic, b) docks and c) mooring areas. Here, the total number of replicates is used as a proxy for study size

	Studies	No. of replicates	Estimate	Lower bound	Upper bound	Std. error	p-val.
**a**	Eriksson	2186	**-0.413**	-0.459	-0.367	0.023	NA
	+ Willby	316	**-0.969**	-2.079	0.141	0.566	0.087
	+ Murphy	105	**-0.910**	-1.721	-0.100	0.414	0.028
	+ Doyle	72	**-0.797**	-1.306	-0.289	0.260	0.002
	+ Mueller	10	**-0.718**	-1.146	-0.289	0.219	0.001
	+ Asplund	10	**-0.593**	-0.888	-0.298	0.151	< 0.001
	+ Vermaat	10	**-0.615**	-0.898	-0.331	0.145	< 0.001
**b**	Gladstone	213	**-1.513**	-1.727	-1.300	0.109	NA
	+ Fyfe	210	**-1.471**	-1.661	-1.280	0.097	< 0.001
	+ Steinmetz	162	**-1.478**	-1.666	-1.290	0.096	< 0.001
	+ Loflin	162	**-2.153**	-3.598	-0.708	0.737	0.004
	+ Burdick	60	**-2.112**	-3.329	-0.895	0.621	< 0.001
	+ Campbell	40	**-1.941**	-3.014	-0.869	0.547	< 0.001
	+ Eriander	26	**-1.856**	-2.815	-0.897	0.489	< 0.001
**c**	Eriksson	3640	**-0.168**	0.211	-0.126	0.022	NA
	+ Hansen	609	**0.058**	-0.399	0.516	0.233	0.802
	+ Marba	280	**-0.124**	-0.479	0.231	0.181	0.492
	+ Nordlund	150	**-0.130**	-0.376	0.115	0.125	0.298
	+ Fernandez-T	34	**-0.188**	-0.415	0.039	0.116	0.105
	+ Mueller	10	**-0.226**	-0.445	-0.007	0.112	0.043

Sensitivity analysis

Sensitivity for choice regarding aggregation of data (Shenhav et al. 2015)

The summary effects of boat traffic, docks and mooring areas remained significant and largely independent of the decision to analyze the data sets per site and survey (as done in the article), or per publication (Figs. S2–S4). When analyzed per publication the summary effect of boat traffic decreased in magnitude from -0.86 to -0.62 (i.e., the impacted sites went from 42 to 54% abundance of the control), while the summary effect for docks instead increased in magnitude from -1.70 to -1.86 (i.e., the impacted sites went from 18 to 16% of the control). However, the estimated summary effects remains within the 95% confidence intervals from the original analyses and the overall interpretation of the results is not affected by the choice of analysis level. For mooring areas the summary effect decreased from -0.57 to -0.23 (i.e., the impacted sites went from 57 to 80% of the control), which is just outside the upper 95%-confidence interval of the original analysis (-0.27).

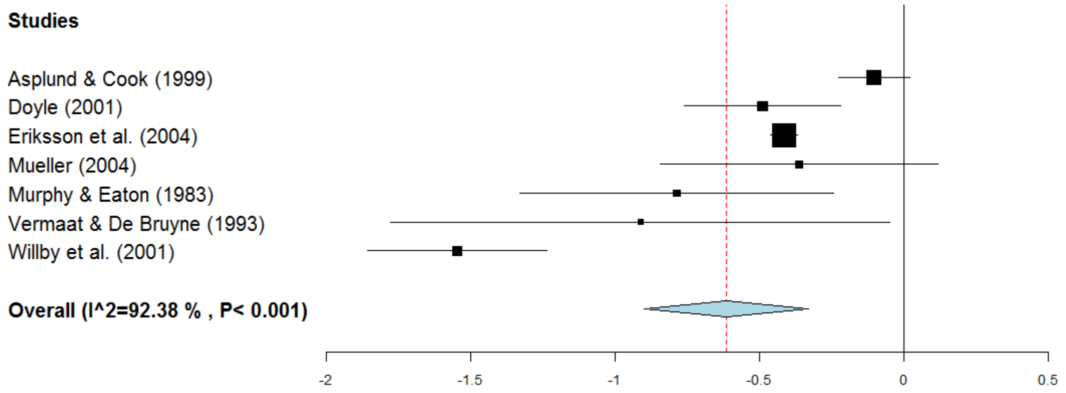

Figure S2. Sensitivity analysis exploring how the summary effect of boat traffic is affected by averaging the effects per study instead of per site and survey. The figure can be compared to Fig. 3 in the main text.

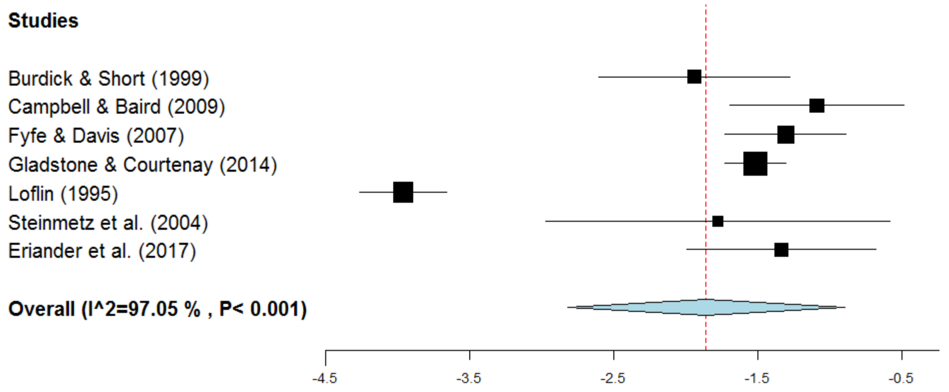

Figure S3. Sensitivity analysis exploring how the summary effect of docks is affected by averaging the effects per study instead of per site and survey. The figure can be compared to Fig. 4 in the main text.

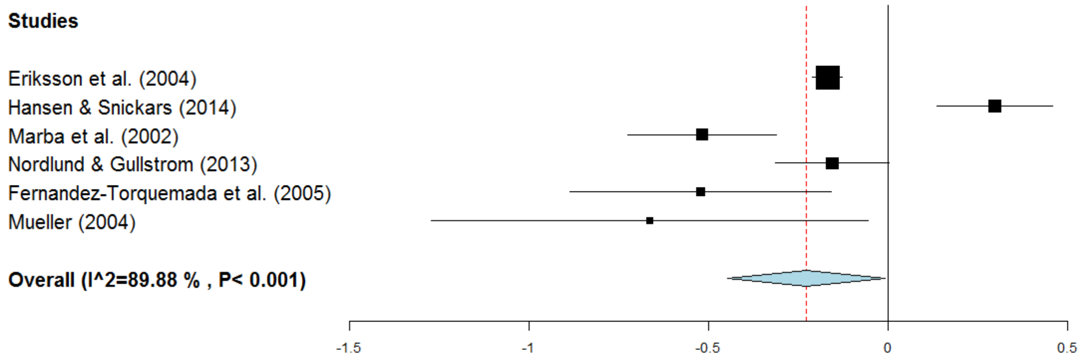

Figure S4. Sensitivity analysis exploring how the summary effect of mooring areas is affected by averaging the effects per study instead of per site. The figure can be compared to Fig. 5 in the main text.

Leave-one-out sensitivity analysis (Shenhav et al. 2015)

The summary effects of boat traffic, docks and mooring areas remained significant and largely independent of the influence of single effect sizes (Figs. S5–S7). In the data set of docks, Loflin (1995) sticks out as a potential outlier (Fig. S6). When excluding it from the analysis the summary effect decreases in magnitude from -1.70 to -1.47 (i.e., the impacted sites went from 18 to 23% of the control). However, the overall interpretation of the results is not affected by the presence or absence of this data point.

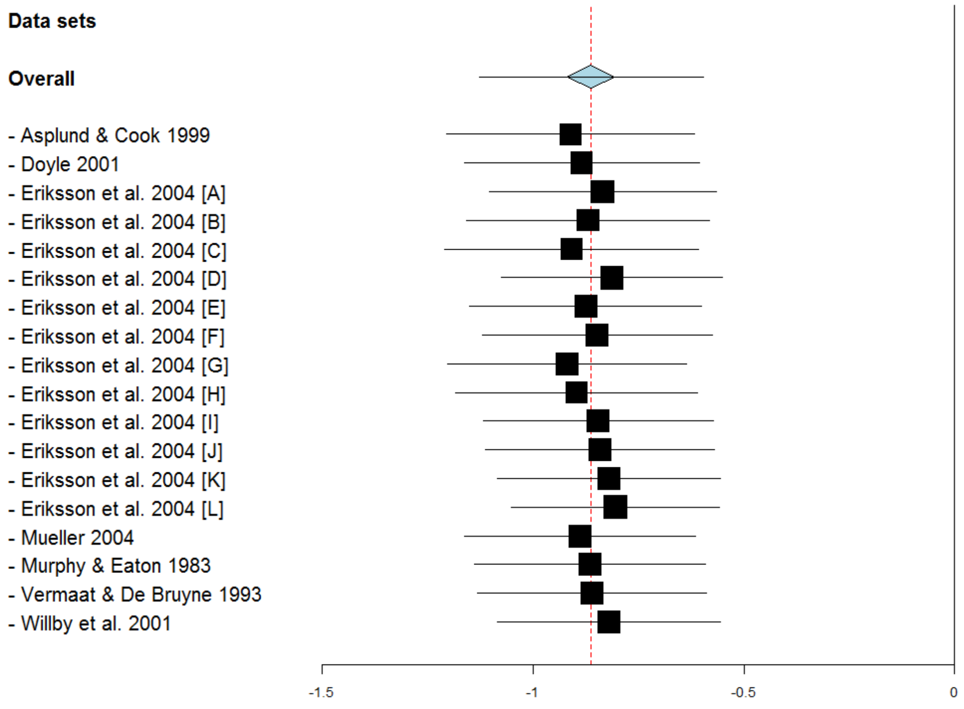

Figure S5. Exploration of how each data point affects the summary effect when removed one at the time with leave-one-out analysis for boat traffic.

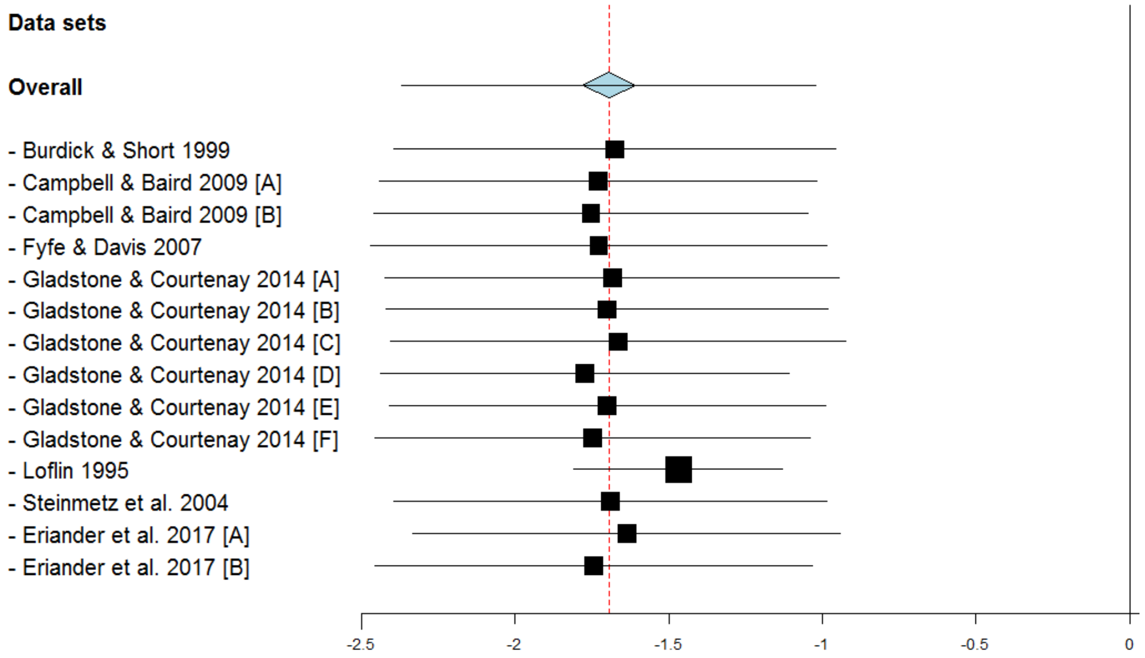

Figure S6. Exploration of how each data point affects the summary effect when removed one at the time with leave-one-out analysis for docks.

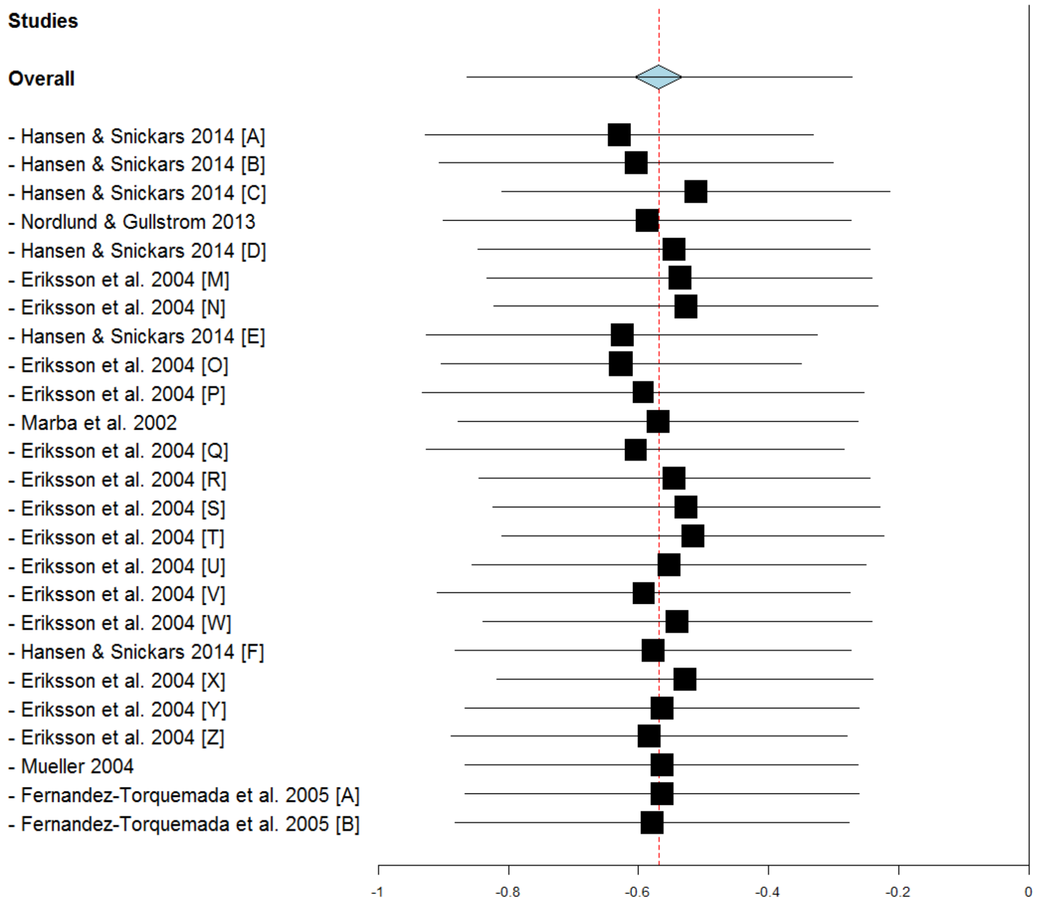

Figure S7. Exploration of how each data point affects the summary effect when removed one at the time with leave-one-out analysis for mooring areas.

References

Borenstein, M., L. V. Hedges, J. Higgins, and H. R. Rothstein. 2009. *Introduction to Meta-Analysis*. West Sussex, UK: Wiley.

Begg, C. B., and M. Mazumdar. 1994. Operating characteristics of a rank correlation test for publication bias. *Biometrics*: 1088–1101.

Koricheva, J., J. Gurevitch, and K. Mengersen. 2013. *Handbook of meta-analysis in ecology and evolution*. Princeton University Press.

Rosenthal, R. 1979. The file drawer problem and tolerance for null results. *Psychological bulletin* 86: 638.

Shenhav, L., R. Heller, and Y. Benjamini. 2015. *Quantifying replicability in systematic reviews: the r-value*. Tecnical report arxiv1502.00088. Technical Report Arxiv1502.00088. Tel-Aviv: Tel-Aviv University.

Appendix S6. Included studies

Table S4 summarize information about the studies included in the review. Table S5 provide an identification key for individual effect sizes from the publications that contributed with more than one effect size.

Table S4. Description of ecological characteristics, the investigated human activity and the extracted data of the included studies of **a**) boat traffic, **b**) docks, **c**) mooring buoys and **d**) mooring areas

	Reference	Characteristics			Description of activity	Description of data
		Measurements	Area and habitat	Depth (m): min–max [mean]		
**a**	Asplund & Cook (1999)	Shoot density (at four point scale) of stoneworts (*Chara* spp.) and milfoils (*Myriophyllum* spp.)	Temperate lake (N USA)	1.0–3.5 [1.5]	The lake is heavily used for recreation, e.g. fishing and water-skiing. It had 16.6 boating days per acre and year, with a maximum of 60 boats present at the same time. One boating day equals one boat being out on the lake at some point during a day	A field experiment comparing an area with temporary boat restriction, with three plots outside of the restricted area (n = 4–6). One effect size extracted
	Doyle (2001)	Biomass (g DW) and number of rooted rosettes of tape grass (*Vallisneria americana*)	Sub-tropic lake (S USA)	0.15–0.25	Artificially created waves repeated five to six times a day, i.e. 20–30 min intense wave action a day within the lower range of boat wakes (0.15 m high)	A mesocosm experiment with tape grass compared after 67 days of growth at 0.15, 0.2 and 0.25 m depth, in a wave raceway and in a control without waves (n = 36, extracted from data of all depths combined). One effect size extracted
	Eriksson et al. (2004)	Cover (%) of mixed vegetation community (e.g. *Ceratophyllum demersum*, *Chara* spp., *Chorda filum*, *Fucus vesiculosus*, *Myriophyllum* spp. *Najas marina*, *Potamogeton* spp., *Ruppia* spp.)	Temperate coast (Sweden)	0.5–5.3 [1.5]	Bays located 120 to 920 m distance from ferry route, navigated daily by 10 to 40 ferryboats (for < 350 passengers, 15–42 m in length according to the operators)	A comparative study comprising 15 impacted bays and 14 control bays, located 2.1–1.2 km from closest ferry rout (n = 24 to 241 depending on bay size). We used raw data provided by the authors in our analysis (see methods section) to generate twelve impact-control pairs (12 effect sizes)
	Mueller (2004)	Shoot density (shoots/m^2) of seagrass (*Halodule wrightii*)	Sub-tropic coast (SE USA)	No data	Area located 400 m from a large marina and adjacent to a high intensity travel route for recreational boats	A comparative study between an impacted seagrass bed and a seagrass bed with little or no disturbance by boats within a nature reserve further out on the coast (n = 5). One effect size extracted

	Reference	Characteristics			Description of activity	Description of data
		Measurements	Area and habitat	Depth (m): min–max [mean]		
	Murphy & Eaton (1983)	Biomass (g DW/m^2) of mixed vegetation community (e.g. *Elodea canadensis*, *Potamogeton* spp., *Nuphar lutea*, *Cladophora glomerata*, *Sparganium erectum*)	Temperate canal system (UK)	0–3	Fifty m canal sections, 8 to 16 m wide and less than 3 m deep, with 89 to 14 000 boat movements per ha per m depth per year (calculated from using the number of times locks were operating). The majority of the boats (81%) were ≤ 9.1 m in length	A comparative study where plant biomass from ca 100 sites along 24 canal stretches is plotted against traffic intensity. The control had no recorded boat movements (n = 7 for the control and 98 for impacted sites). One effect size extracted
	Vermaat & De Bruyne (1993)	Above and below ground biomass, biomass of tubers (mg AFDW), number of shoots and bundles of leaves of pondweed (*Stuckenia pectinata*)	Temperate river (Netherlands)	[0.6]	On average 75 boats a day passing through the 34 m wide river section during the experimental period	A field experiment with BACI-design, where potted pondweed were compared with and without the shelter of a breakwater, within a busy section of a lowland river (n = 4–6). Two out of five outcome measures had to be dropped when calculating the average effect size (i.e., no. of shoots and no. of bundles), since there were no means to calculate how they correlated with the other three outcome measures. One effect size extracted
	Willby et al. (2001)	Biomass (g DW/m^2) of mixed vegetation community (e.g. *Potamogeton* spp., *Lemna* spp., *Cladophora glomerata*, *Elodea* spp., *Callitriche* spp., *Ceratophyllum demersum*, *Nuphar lutea*)	Temperate canal system (UK)	[0.8–1.2]	150 m canal sections, 10 to 15 m wide and on average 0.8 to 1.2 m deep, with 0.05 to 16 500 boat movements per 10 m cross-sections per year (calculated from using the number of times locks were operating)	A comparative study where plant biomass from ca 400 sites distributed randomly over the canal network is plotted against traffic intensity The control had no recorded boat movements (n = 23 for the control and 316 for impacted sites). One effect size extracted
**b**	Burdick & Short (1999)	Shoot density (shoots/m^2) and canopy structure (cm shoots/m^2) of seagrass (*Zostera marina*)	Temperate coast (NE USA)	No data	Shading by floating and fixed private docks, with varying cardinal direction, width (0.7–6.9 m) and deck height above bottom (77–340 cm)	A comparative study comprising samples taken underneath a total of 21 docks and control samples taken perpendicular to each dock at a location away from possible dock impacts (n = 47). The docks were located in four different bays. One effect size extracted

Reference	Characteristics			Description of activity	Description of data
	Measurements	Area and habitat	Depth (m): min–max [mean]		
Campbell & Baird (2009)	Shoot density (stems/m^2) of mixed vegetation community (e.g. *Ceratophyllum demersum*, *Vallisneria americana*, *Potamogeton illinoensis*, *Utricularia foliosa*, *Nitella* spp.)	Sub-tropical lake (SE USA)	0.1–1.7	Shading by fixed private docks, with varying cardinal direction, width (2.6–4.9 m), deck height above water (0.57–1.30 m) and plank spacing (0–14 mm)	A comparative study comprising 20 docks from two lakes (n = 10). The control samples were located at undisturbed shoreline near surveyed docks (< ca 50 m) or at a representative site with undisturbed shoreline further away from the dock. Two effect sizes extracted
Eriander et al. (2017)	Vegetation cover (%) of seagrass (*Zostera marina*)	Temperate coast (Sweden)	0.2–3.8 [1.2]	Shading by floating and fixed docks, with varying length (7–162 m), width (0.8–3.1 m) and height above water (0–1.7 m).	A comparative study comprising fixed docks (n = 10, with 9 controls) and floating docks (n = 4, with 3 controls) from four different coastal areas. The control samples were located 6 m from the dock edge, perpendicular to the length axis of the docks. Each replicate comprised of a mean value of 6 measurements taken along the length axis of the docks. Two effect sizes extracted
Fyfe & Davis (2007)	Shoot density (shoots/0.25m^2) seagrass (*Posidonia australis*)	Sub-tropic coast (SE Australia)	2–4	Shading by fixed public dock, constructed by open aluminium mesh. Deck width and height above water not reported	A comparative study with BACI-design, of the impact by the construction of a 75 m long pier stretching over a seagrass bed. We have used the samples from the seagrass bed 50 m from the construction as control (n = 20 for the control and 10 for samples taken under dock). One effect size extracted
Gladstone & Courtenay (2014)	Total biomass (gDW/0.008m^2) seagrass (*Zostera muelleri*)	Sub-tropic coast (SE Australia)	≤1	Shading by fixed private docks made from wood or aluminium mesh, with either east–west or north-south orientation. Deck width 0.9–1.1 m, deck height above water 0.6–0.7 m and plank spacing if applicable 10–12 mm	From four comparative studies exploring different variables affecting dock impact, i.e., cardinal direction (n = 15), difference in impact between wood and mesh docks (n = 7), and two BACI studies of dock installations (a wooden and a mesh dock, n = 5 for the docks and 30 for the controls). The control samples of all studies were taken at seagrass beds 250 m to 5 km from the docks. Six effect sizes extracted

	Reference	Characteristics			Description of activity	Description of data
		Measurements	Area and habitat	Depth (m): min–max [mean]		
	Loflin (1995)	Shoot density (shoots/0.01m^2) seagrass (*Halodule wrightii* and *Syringodium filiforme*)	Sub-tropic coast (SE USA)	No data	Shading by fixed private docks. Dock orientation varied as well as dock width (1.3–2.1 m) and deck height above water (0.65–1.45 m)	A comparative study comprising 27 docks from 4 different sites. Three samples were taken under each dock and 3 control samples on the non-shaded bottom adjacent to each dock (n = 81). No variance measures are reported. One effect size extracted
	Steinmetz et al. (2004)	Vegetation cover (%) of mixed plant community (primarily *Vallisneria americana*)	Sub-tropical river (SE USA)	[0.6–0.7]	Shading by fixed, wooden experimental docks, with no spacing, oriented in east–west direction. The docks were 1.5 m wide and the deck 0.9 m above the water surface	A field experiment with BACI-design, were standardized docks were placed in a vegetated area 50 m from the shore. Docks with and without glass prisms underneath were compared to controls taken in plant beds without docks (n = 3). One effect size extracted
**c**	Colomer et al. (2017)	Shoot density and vegetation cover (%) of seagrass (*Posidonia oceanica*)	Sub-tropic coast (Spain)	7.3–8.7	Physical damage by conventional swing moorings	A comparative study of the past impact from three swing moorings in one site. The size of the vegetation-free gap in vegetation was measured and the vegetation was sampled close to the gap and compared to a control area in vegetation at similar depth (n = 4–10)
	Demers et al. (2013)	Shoot density (% of maximum at reference) and vegetation cover (%) of seagrass (*Posidonia australis*)	Sub-tropic coast (SE Australia)	3–6	Physical damage by three mooring types, i.e., seagrass-friendly screw moorings, conventional swing moorings and cyclone moorings	A comparative study of four screw moorings, two swing moorings and three cyclone moorings. The vegetation was sampled at 0–2, 3–5 and 6–8 m from the centre of the swing zone and compared to nearby reference areas (n = 4 for shoot density and 2 for vegetation cover per sampling distance and buoy/reference)
	Glasby and West (2018)	Vegetation cover (%) of seagrass (*Posidonia australis*)	Sub-tropic coast (SE Australia)	0.4–5.6	Physical damage by conventional swing moorings	A comparative study of the impact of 30 swing moorings in two sites. The vegetation cover was estimated for the entire scarred area and compared with adjacent control plots outside the mooring scars

Reference	Characteristics			Description of activity	Description of data
	Measurements	Area and habitat	Depth (m): min–max [mean]		
La Manna et al. (2015)	Shoot density (shoots/m^2) of seagrass (*Posidonia oceanica*)	Sub-tropic coast (Italy)	3–16	Physical damage by conventional swing moorings	A comparative study of the impact from ten swing moorings from two sites. The vegetation was sampled at 0–3 and 6–9 m from centre of the swing zone and compared to control plots in vegetation at similar depth (n = 5)
Montefalcone et al. (2008)	Shoot density (shoots/m^2) of seagrass (*Posidonia oceanica*)	Sub-tropic coast (Italy)	3–12	Physical damage by an 1130 m long chain system, running along the bottom between anchor points	A comparative study with BACI-design, of a seagrass bed before buoy chains were deployed in spring and when the chains were retrieved for the winter. The vegetation was sampled within and outside of the reach of the chain. The experimental design is hierarchical with samples taken both in the deep and shallow part of the bed and in areas with high, medium and low cover of seagrass (n = 6)
Ostendorp et al. (2009)	Cover (%) of Stonewort-dominated vegetation (*Chara* spp.)	Temperate lake (Germany)	0.9–1.7	Physical damage by conventional swing moorings and vegetation-friendly hook-buoys. The municipal manager adjusts the chains of the hook-buoys on a weekly basis according to the current water level, while the conventional buoys has a long chain to account for high water levels during summer.	A comparative study of the impact from six swing moorings and six hook-buoys, compared to unaffected areas with a perimeter of 3 to 5 m outside of each impacted area (n = 6). The sampling method is poorly described
Unsworth et al. (2017)	Vegetation cover (%) of seagrass (*Zostera marina*)	Temperate coast (UK)	No data	Physical damage by conventional swing moorings	A comparative study of swing moorings in five sites, compared with control areas in the same meadows. The vegetation was sampled every two meters along two 20 m transects through the centre of the swing zone

	Reference	Characteristics			Description of activity	Description of data
		Measurements	Area and habitat	Depth (m): min–max [mean]		
**d**	Eriksson et al. (2004)	Cover (%) of mixed vegetation community (e.g. *Ceratophyllum demersum*, *Chara* spp., *Chorda filum*, *Fucus vesiculosus*, *Myriophyllum* spp. *Najas marina*, *Potamogeton* spp., *Ruppia* spp.)	Temperate coast (Sweden)	0.5–5.0 [1.6]	Bays with docks for recreational boats (ca. 5–7 m in length). The total number of berths ranged from 30 to 250 per bay	A comparative study comprising 15 mooring bays and 14 control bays with <5 berths, situated >800 m from boating routes and not utilized as popular anchoring sites (n = 24 to 391 depending on bay size). We used raw data provided by the authors in our analysis (see methods section) to generate 14 impact-control pairs (14 effect sizes)
	Fernandez-Torquemada et al. (2005)	Vegetation cover (%) and shoot density (shoots/m2) of seagrass (*Posidonia oceanica*)	Sub-tropic coast (Spain)	[5]	Two large size marinas with a total of 542 and over 862 berths respectively	A comparative study were seagrass beds adjacent to two large marinas have been compared to two reference sites with similar environmental conditions (n = 3). Sampling method and the control area are poorly described. Two effect sizes extracted
	Hansen & Snickars (2014)	Cover (%) of mixed vegetation community (e.g. *Ceratophyllum demersum*, *Chara* spp., *Fucus vesiculosus*, *Myriophyllum* spp. *Najas marina*, *Potamogeton* spp., *Ruppia* spp., *Zannichellia palustris*)	Temperate coast (Sweden)	0.5–3.9 [1.0]	Bays with docks for recreational boats (5–12 m in length according to unpublished data provided by authors). The total number of berths ranged from 12 to 180 per bay	A comparative study comprising six mooring bays and six control bays with <5 berths, situated >800 m from boating routes, and not utilized as popular anchoring sites (n = 8 to 171 depending on bay size). We used raw data provided by the authors in our analysis (see methods section) to generate six impact-control pairs (6 effect sizes)
	Marbá et al. (2002)	Vegetation cover (%) and shoot density (shoots/m^2) of seagrass (*Posidonia oceanica*)	Sub-tropic coast (Spain)	0-40	A bay with a buoy field of 50 fixed mooring points and a small ferry service , docking at the pier a couple of times a day during summer	A comparative study where a mooring bay is compared with a control bay nearby that has been closed to visitors for seven years. The vegetation cover was monitored with an echo sounder at five depths (n = 20) and the shoot density was sampled at four to seven depths depending on site (n = 10). One effect size extracted

Reference	Characteristics			Description of activity	Description of data
	Measurements	Area and habitat	Depth (m): min–max [mean]		
Mueller (2004)	Shoot density (shoots/m^2) of seagrass (*Halodule wrightii*)	Sub-tropic coast (SE USA)	No data	A high and dry boat marina with approximately 396 boat mooring facilities (estimated by us, see methods section)	A comparative study between a seagrass bed adjacent to a large marina and a seagrass bed with little or no disturbance by boats within a nature reserve further out on the coast (n = 5). One effect size extracted
Nordlund & Gullström (2013)	Shoot density (shoots/m^2), above and below ground biomass (g DW/m^2) of seagrass (primarily *Thalassodendron ciliatum*)	Sub-tropic coast (Mozambique)	No data	A bay with an average of 26 motor and sailing boats docked at pier and at moorings in the intertidal zone. The hotel located adjacent to the harbour offered daily water sport activities	A comparative study where a mooring area is compared to a reference site within a nature reserve across the bay, 2 km from the impacted site (n = 25). One effect size extracted

Table S5. Identification key that links individual datasets to their source. Only datasets from publications that has contributed to more than one data point are included

Data category	Publication	Index	Data sets
Traffic and wake	Eriksson et al. 2004	A	Impacted bay N59.4574 E18.7346, control bay N59.3298 E18.7404
	Eriksson et al. 2004	B	Impacted bay N59.4756 E18.8209, control bay N59.048 E18.188
	Eriksson et al. 2004	C	Impacted bay N59.0836 E18.4813, control bay N59.1125 E18.5094
	Eriksson et al. 2004	D	Impacted bay N59.4491 E18.7388, control bay N59.1079 E18.6697
	Eriksson et al. 2004	E	Impacted bay N59.421 E18.6556, control bay N59.3489 E18.7378
	Eriksson et al. 2004	F	Impacted bay N59.4282 E18.6363, control bay N59.4177 E19.0143
	Eriksson et al. 2004	G	Impacted bay N59.6644 E18.783, control bay N59.7741 E19.1665
	Eriksson et al. 2004	H	Impacted bay N59.4809 E18.8161, control bay N59.1169 E18.5222
	Eriksson et al. 2004	I	Impacted bay N59.2987 E18.787, control bay N59.4529 E18.8058
	Eriksson et al. 2004	J	Impacted bay N59.4538 E18.8231, control bay N59.0199 E18.5529
	Eriksson et al. 2004	K	Impacted bay N59.494 E18.8518, control bay N59.4186 E18.9386
	Eriksson et al. 2004	L	Impacted bay N59.4091 E18.6121, control bay N59.1199 E18.3713
Docks	Campbell & Baird 2009	A	Docks vs. control sites in Lake Butler N28.4862 W81.5537
	Campbell & Baird 2009	B	Docks vs. control sites in Lake Jessamine N28.4820 W81.3850
	Gladstone & Courtenay 2014	A	Docks with cardinal direction E-W vs. control sites in Lake Macquarie S33.0546 E151.6065
	Gladstone & Courtenay 2014	B	Docks with cardinal direction N-S vs. control sites in Lake Macquarie S33.0546 E151.6065
	Gladstone & Courtenay 2014	C	Wooden docks vs. control sites in Lake Macquarie S33.0546 E151.6065
	Gladstone & Courtenay 2014	D	Mesh docks vs. control sites in Lake Macquarie S33.0546 E151.6065
	Gladstone & Courtenay 2014	E	Wooden docks before-and-after-impact (BACI) in Lake Macquarie S33.0546 E151.6065
	Gladstone & Courtenay 2014	F	Mesh docks BACI in Lake Macquarie S33.0546 E151.6065
	Eriander et al. 2017	A	Floating docks[1] vs contols adjecent to dock in 4 areas on the NW coast of Sweden N58.2951 E 11.5046
	Eriander et al. 2017	B	Fixed docks vs contols adjecent to dock in 4 areas on the NW coast of Sweden N58.2951 E 11.5046

[1] Response ratios were calculatde using cover recorded at the edge of the docks, since the cover under docks was zero.

Table S5. continued

Data category	Publication	Index	Data sets
Mooring areas	Hansen & Snickars 2014	A	Impacted bay N56.1023 E15.6119, control bay N56.1513 E15.1157
	Hansen & Snickars 2014	B	Impacted bay N60.0773 E20.2215, control bay N60.1047 E20.2732
	Hansen & Snickars 2014	C	Impacted bay N60.8921 E17.2292, control bay N60.7749 E17.291
	Hansen & Snickars 2014	D	Impacted bay N60.0934 E20.2644, control bay N60.0612 E20.4804
	Hansen & Snickars 2014	E	Impacted bay N60.121 E20.2577, control bay N60.0172 E20.5043
	Hansen & Snickars 2014	F	Impacted bay N58.6145 E16.9189, control bay N58.5429 E16.8133
	Eriksson et al. 2004	M	Impacted bay N59.5345 E18.7237, control bay N59.048 E18.188
	Eriksson et al. 2004	N	Impacted bay N59.1134 E18.4788, control bay N59.7741 E19.1665
	Eriksson et al. 2004	O	Impacted bay N59.3362 E18.7017, control bay N59.2799 E18.5584
	Eriksson et al. 2004	P	Impacted bay N59.3534 E18.6253, control bay N 59.1125 E 18.5096
	Eriksson et al. 2004	Q	Impacted bay N59.2994 E18.6339, control bay N59.6641 E18.869
	Eriksson et al. 2004	R	Impacted bay N59.2812 E18.6907, control bay N59.1169 E18.5222
	Eriksson et al. 2004	S	Impacted bay N59.2622 E18.5511, control bay N59.3298 E18.7404
	Eriksson et al. 2004	T	Impacted bay N59.5366 E18.7484, control bay N59.3489 E18.7378
	Eriksson et al. 2004	U	Impacted bay N59.4575 E18.5971, control bay N59.0199 E18.5529
	Eriksson et al. 2004	V	Impacted bay N59.35 E18.6635, control bay N59.1199 E18.3713
	Eriksson et al. 2004	W	Impacted bay N59.2831 E18.6311, control bay N59.3979 E18.7375
	Eriksson et al. 2004	X	Impacted bay N59.504 E18.6392, control bay N59.1079 E18.6697
	Eriksson et al. 2004	Y	Impacted bay N59.3825 E18.5301, control bay N59.494 E18.8518
	Eriksson et al. 2004	Z	Impacted bay N59.276 E18.5384, control bay N59.2987 E18.787
	Fernandez-Torquemada et al. 2005	A	Marina Luis Campomamanes (N38.6292 W0.0038) vs. nearby control site
	Fernandez-Torquemada et al. 2005	B	Port of Altea (N38.5884 W0.0543) vs. nearby control site

Appendix S7. Meta regressions

Meta-regression of vegetation abundance against the number of berths (Fig. S8, coefficient = -0.000, SE = 0.001, Z = -0.082, p = 0.93) and the number of berths per ha (Fig. S9, coefficient = -0.003, SE = 0.007, Z = -0.396, p = 0.69). The test for residual heterogeneity was significant for both models (I^2 = 98%, T^2 = 0.507, p < 0.001 and I^2 = 98%, T^2 = 0.561, p < 0.001, respectively).

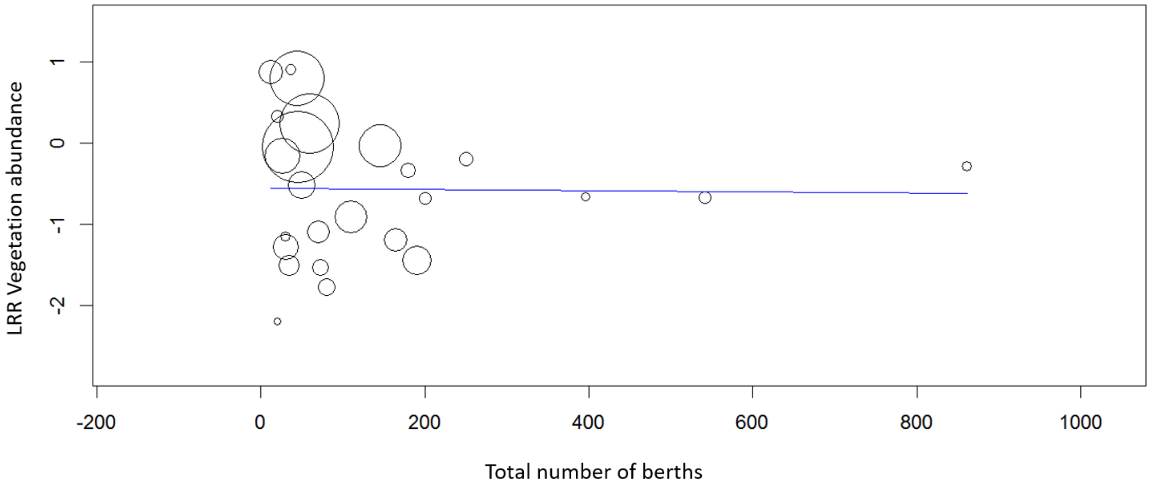

Figure S8. Meta-regression of vegetation abundance against the number of berths per mooring area. Each circle represents one study and size of the circle indicates the study weight.

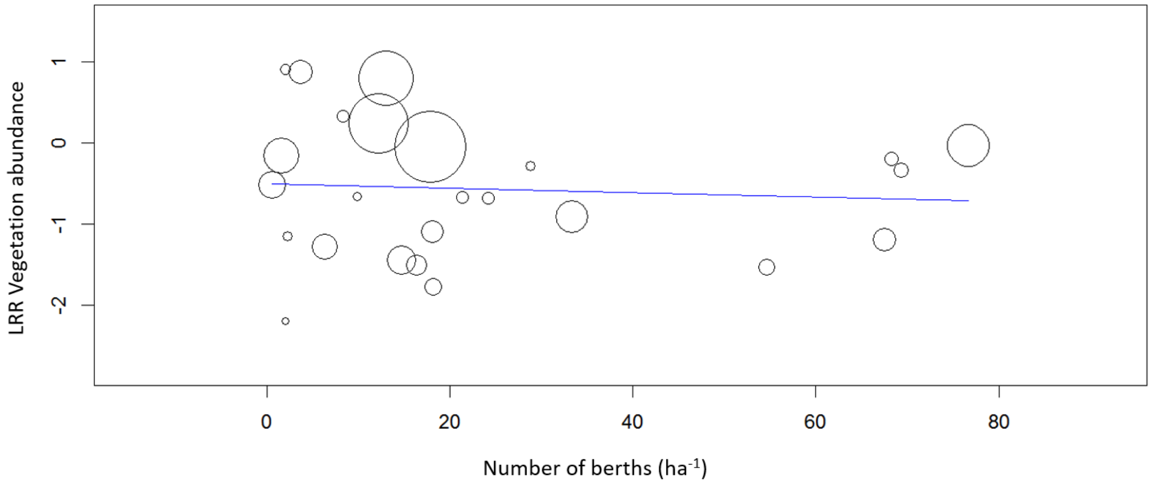

Figure S9. Meta-regression of vegetation abundance against berths per hectare. Each circle represents one study and size of the circle indicates the study weight.
